# Psychrotolerant antarctic *Mokoshia mucilaginosa* and *Mokoshia rubra* enhance salt Stress tolerance in *Nicotiana tabacum* via photosynthetic stabilization and antioxidant regulation

**DOI:** 10.1007/s00299-026-03732-w

**Published:** 2026-02-21

**Authors:** Syed Inzimam Ul Haq, Josef Hájek, Davide Giordano, Ivana Mašlaňová, Ivo Sedláček, Miloš Barták

**Affiliations:** 1 Department of Experimental Biology, Faculty of Science, Laboratory of Photosynthetic Processes, Masaryk University, Kamenice 5, 625 00 Brno, Czech Republic; 2 Department of Experimental Biology, Faculty of Science, Section of Genetics and Molecular Biology, Masaryk University, 611 37 Brno, Czech Republic; 3 Department of Experimental Biology, Faculty of Science, Czech Collection of Microorganisms, Masaryk University, 625 00 Brno, Czech Republic

**Keywords:** Antarctic bacteria, Mokoshia rubra, Mokoshia mucilaginosa, Salinity stress, Photosynthetic efficiency, Antioxidant defense., Bioinoculants., Nicotiana tabacum

## Abstract

**Key message:**

**Antarctic Mokoshia mucilaginosa and Mokoshia rubra are associated with improved salinity tolerance in Nicotiana tabacum through coordinated regulation of photosynthetic performance and antioxidant responses, highlighting the potential of polar bacteria as bioinoculants for sustainable agriculture in saline and cold-affected ecosystems.**

**Abstract:**

Soil salinity is an escalating global challenge that constrains crop productivity worldwide, with particularly severe impacts in marginal agroecosystems, including those in cold regions. Here, we provide evidence that two psychrotolerant Antarctic bacterial strains, *Mokoshia mucilaginosa* and *Mokoshia rubra*, function as plant growth–promoting bioinoculants that alleviate NaCl-induced stress in *Nicotiana tabacum*. Both strains exhibited key plant growth–promoting traits, including indole-3-acetic acid production, phosphate solubilization, siderophore production, and nitrogen fixation. Under salinity levels of 50–150 mM NaCl, bacterial inoculation was associated with improved plant performance, including enhanced biomass accumulation, improved photosystem II efficiency (Fv/Fm, Φ_PSII_, PI_ABS_), and increased pigment contents (chlorophylls and carotenoids), alongside modulation of antioxidant enzyme activities (SOD, POD, CAT, and APX). Fluorescence kinetics and spectral reflectance indices further revealed distinct multivariate patterns separating inoculated plants from uninoculated salt-stressed controls. Together, these results suggest that Antarctic *Mokoshia* spp. contribute to improved photosynthetic function and redox regulation under salinity stress. To our knowledge, this study provides the first report linking members of the genus *Mokoshia* with enhanced salt stress tolerance in plants, highlighting their potential as sustainable microbial tools for improving crop performance in saline agroecosystems.

**Graphical abstract:**

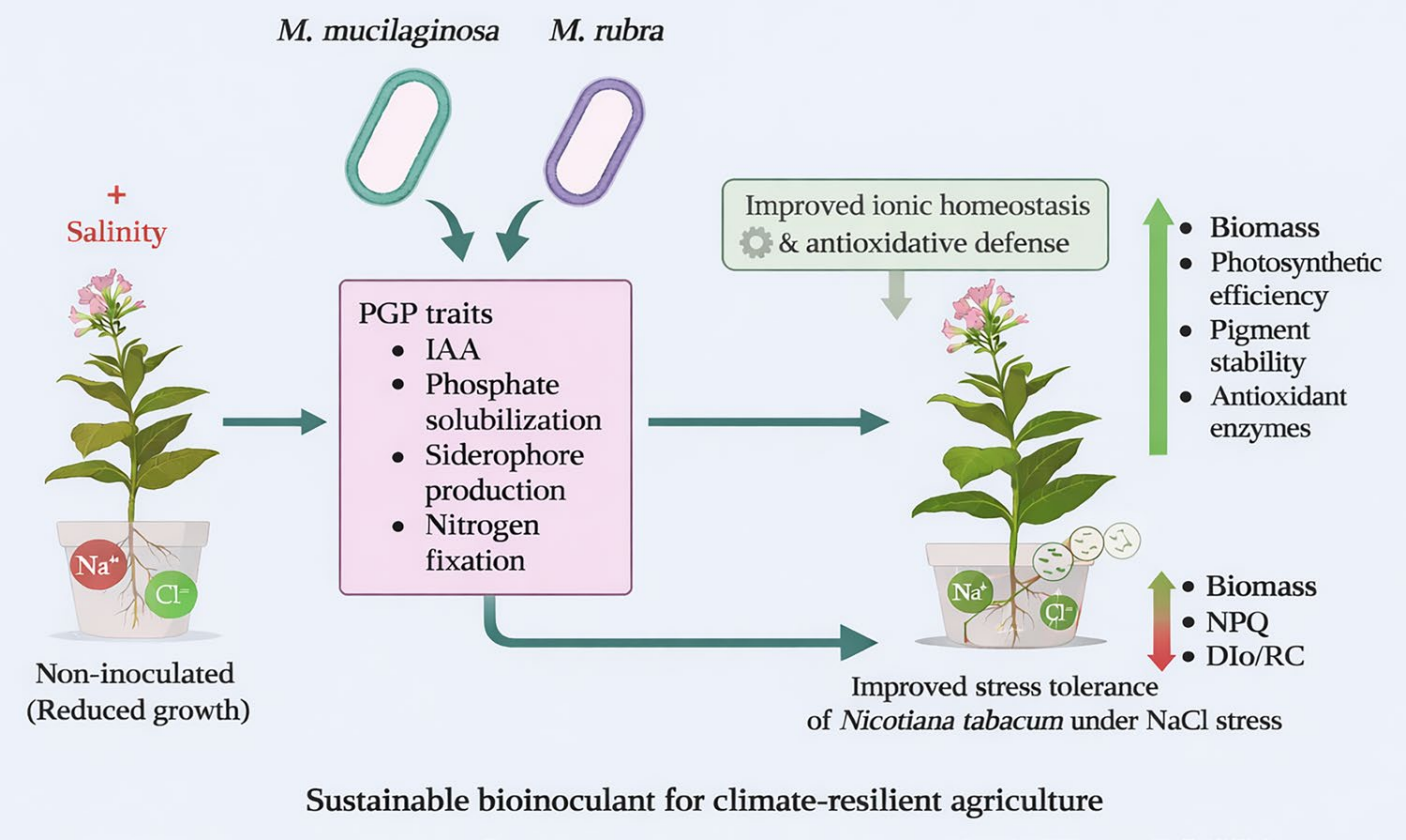

**Supplementary Information:**

The online version contains supplementary material available at 10.1007/s00299-026-03732-w.

## Introduction

Soil salinization represents a major and escalating challenge to global agriculture. It currently affects more than 424 million hectares of topsoil and 833 million hectares of subsoil worldwide, with projections of further expansion under climate change scenarios (Shokri et al. 2024). Salinity simultaneously triggers osmotic, ionic, and oxidative stress, disrupting nutrient uptake, photosynthesis, and cellular homeostasis, and ultimately causing severe reductions in plant growth and crop yield (Balasubramaniam et al. 2023; Islam et al. 2024; Mishra et al. 2023). Although genetic engineering and conventional breeding approaches have been applied to improve salt tolerance, these strategies are laborious, costly, and often deliver limited success under variable field conditions. Consequently, there is growing interest in sustainable alternatives, particularly the use of plant growth-promoting bacteria (PGPB), which improve stress resilience through multiple physiological and biochemical mechanisms (Xie et al. 2024; Gómez-Godínez et al. 2023; Valencia-Marin et al. 2025). PGPB enhance plant performance under abiotic stress via diverse traits such as phosphate solubilization, nitrogen fixation, indole-3-acetic acid (IAA) synthesis, and production of siderophores and antioxidant metabolites. Collectively, these mechanisms promote root growth, nutrient acquisition, redox balance, and stress tolerance (Kour & Yadav 2023; Li et al. 2021; Rizvi et al. 2021). Psychrotolerant PGPB from extreme habitats, including the Andes, Arctic, and Antarctic, are particularly promising because they are pre-adapted to low temperatures, nutrient limitation, and oxidative stress (Araya et al. 2020; Vega-Celedón et al. 2021; Yarzábal et al. 2018). In Antarctica, bacterial communities associated with native plants such as *Deschampsia antarctica* exhibit traits including cold tolerance, ACC deaminase activity, nitrogen metabolism, and IAA production, which enhance plant resilience under multiple stresses (Cid et al. 2018; Iungin et al. 2024). However, Antarctic-derived bacterial genera remain underexplored for their biotechnological potential in agriculture. The genus *Mokoshia* (recently reclassified from Massilia) includes species well adapted to extreme environments such as Antarctic soils (Bowman 2023; Holochová et al. 2020). *M*. *mucilaginosa* and *M*. *rubra*, isolated from James Ross Island, harbor genetic traits linked to oxidative stress tolerance, auxin biosynthesis, nitrogen metabolism, and heavy-metal resistance, suggesting potential roles in plant stress mitigation (Holochová et al. 2020). Other members of the group, including *M*. *phosphatilytica* and *Massilia* sp. ONC3, are reported to enhance phosphate solubilization and facilitate plant symbioses (Peta et al. 2021; Zheng et al. 2017). *Mokoshia* spp. occur across diverse ecological niches, from polar regions to temperate soils and freshwater habitats, highlighting their ecological versatility and potential as bioinoculants (Bowman 2023; Ofek et al. 2012). Despite this, no study has systematically evaluated the role of *Mokoshia* spp. in conferring salt tolerance to plants. To address this gap, we selected *Nicotiana tabacum* (tobacco) as a model host. Tobacco is highly sensitive to salt stress, which disrupts ionic homeostasis, pigment stability, photosynthetic efficiency, and redox balance (Pecherina et al. 2022; Shirokikh et al. 2022), making it an ideal system for assessing the protective effects of PGPB. We hypothesized that the Antarctic genus *Mokoshia*, represented by the psychrotolerant strains *M*. *mucilaginosa* and *M*. *rubra*, can act as novel bioinoculants by mitigating NaCl stress in *N*. *tabacum*. Specifically, we hypothesized that inoculation would be associated with improved biomass accumulation, photosynthetic performance, pigment stability, and modulation of antioxidant responses under salinity stress. Accordingly, this study systematically evaluates the physiological, biochemical, and spectral responses of *N*. *tabacum* to bacterial inoculation under salinity, providing, to our knowledge, the first evidence that *Mokoshia* spp. can support plant growth under saline and cold-prone conditions, with direct implications for sustainable agriculture.

## Materials and methods

### *Mokoshia* strains: source and cultivation

*M*. *rubra* (CCM 8692^ T^) and *M*. *mucilaginosa* (CCM 8733^ T^) were obtained from the Czech Collection of Microorganisms (CCM), Brno, Czech Republic (http://www.sci.muni.cz/ccm/). Both strains were originally isolated from environmental samples collected on the Ulu Peninsula, James Ross Island, Antarctica, as previously described by Holochová et al. (2020), who also provided comprehensive phenotypic, genotypic, and whole-genome sequence data. For cultivation, bacterial strains were grown in Reasoner's 2A (R2A) broth at 28 °C under continuous agitation at 150 rpm. Cultures were incubated for 48 h to reach the logarithmic growth phase. Cell suspensions were subsequently standardized to an optical density at 600 nm (OD₆₀₀) of 0.8–1.0, corresponding to approximately 1 × 10⁸ CFU/mL, as measured using a spectrophotometer.

### NaCl tolerance and plant growth-promoting potential of *Mokoshia* Strains

*Mokoshia* spp. strains were evaluated for salt tolerance by exposing them to increasing concentrations of NaCl. For salt tolerance assessment, bacterial cultures were grown in R2A broth at 28 °C with shaking (150 rpm) until they reached an initial optical density of OD₆₀₀ ≈ 0.1. Salinity stress was imposed by supplementing the growth medium with NaCl to final concentrations of 0, 50, 100, 150, 200, and 250 mM prior to inoculation. Bacterial growth under saline conditions was monitored by measuring OD₆₀₀ at defined time intervals. The plant growth–promoting potential of *M*. *rubra* and *M*. *mucilaginosa* was evaluated through standard qualitative assays. Indole-3-acetic acid (IAA) production was quantified following Sarwar and Kremer (1995) using L-tryptophan–supplemented R2A broth; the development of a pink coloration after addition of Salkowski reagent indicated IAA presence, and absorbance was measured at 530 nm. Phosphate solubilization was determined on Pikovskaya's agar (Gaur 1990), where clear halo zones around bacterial colonies indicated solubilization activity. Siderophore production was assayed using Chrome Azurol S (CAS) agar following Alexander and Zuberer (1991); an orange halo surrounding the colonies signified siderophore secretion. Nitrogen fixation ability was assessed on nitrogen-free semi-solid malate medium according to Baldani et al. (2014). Growth and pellicle formation near the medium surface after incubation confirmed N2-fixation potential (see Supplementary Materials, Table 1).

### Plant growth experiment

Uniform, healthy seeds of *N*. *tabacum* (tobacco) were surface sterilized by immersion in 70% (v/v) ethanol for 1 min, followed by 10% (v/v) commercial bleach solution (≈ 0.5% sodium hypochlorite) for 15 min, and rinsed five times with sterile deionized water to remove residual disinfectant. Sterilized seeds were germinated in autoclaved sand under controlled environmental conditions. For bacterial inoculation, *Mokoshia* spp. cell suspensions (10⁸ CFU/mL) were applied to autoclaved soil by adding 20 mL of inoculum per pot (containing 500 g of soil). The inoculated soil was left to equilibrate for 24 h prior to transplantation. Tobacco seedlings at the two-leaf stage were then transplanted individually into each pot. Salt stress was imposed 10 days post-transplantation by supplementing the soil with NaCl at concentrations of 50, 100, and 150 mM. Control pots received an equivalent volume of sterile demineralized water. All treatments were conducted in a greenhouse under controlled conditions: 40% relative humidity, a 16-h light/8-h dark photoperiod, and a temperature of 23 ± 3 °C. The experiment followed a completely randomized design (CRD) with five biological replicates per treatment. A factorial arrangement was employed to examine the interaction between two independent factors: NaCl stress levels and bacterial inoculation.

### Plant biomass determination

At the end of the experimental period, plants were carefully harvested, and biomass was assessed by measuring both fresh and dry weights (30 days after salt stress imposition). Whole plants were carefully harvested, and total fresh weight was recorded immediately, using a calibrated digital balance. For dry weight determination, plant samples were oven-dried at 65 °C until a constant weight to determine total dry biomass.

### Chlorophyll fluorescence, ojip transients, and spectral reflectance measurements

Chlorophyll fluorescence parameters were measured on fully expanded leaves after dark adaptation using a FluorCam FC 800 system (Photon Systems Instruments, Czech Republic), following standard protocols to assess PSII efficiency and energy flux dynamics. For fluorescence imaging, leaves were selected from comparable positions on each plant (fully expanded, non-senescent leaves), although leaf size varied among treatments because of salinity-induced growth inhibition. Slow Kautsky kinetics (KK), complemented by quenching analysis, were performed following the method described by Barták et al. (2023). Key chlorophyll fluorescence parameters, including maximum quantum yield [Fv / Fm = Fm − F_0_ / Fm] (Genty et al. 1989), effective quantum yield [Φ_PSII_ = F′m − Fs / F′m] (Genty et al. 1989), photochemical quenching coefficient [qP = F′m – Fs / F′m − F′_0_] (Krause & Weis 1991), non-photochemical quenching [NPQ = Fm − F′m / F′m] (Müller et al. 2001), and chlorophyll fluorescence decrease ratio [RFD = Fp – Fs / Fs] (Lichtenthaler et al. 2005), were recorded to evaluate photosynthetic efficiency.

To investigate the effects of NaCl stress and plant growth-promoting bacteria (PGPB) on the primary photochemical events in photosystem II (PSII), fast chlorophyll fluorescence transients (OJIP) were measured using a FluorPen FP 110 fluorometer (Photon Systems Instruments, Czech Republic). Leaves were dark-adapted for 10 min prior to measurement. OJIP parameters including performance index on absorption basis: PI_ABS_ = (RC/ABS) × [(φP_0_ / 1—φP_0_)] × [(ψ_0_ / 1—ψ_0_)], electron transport flux per reaction center: ETo/RC = M_0_ × (1 / V_J_) × ψ_0_, trapped energy flux per reaction center: TRo/RC = M_0_ × (1 / V_J_), absorbed energy flux per reaction center: ABS/RC = M_0_ × (1 / V_J_) × (1 / φP_0_), and dissipated energy flux per reaction center: DIo/RC = (ABS/RC) – (TR_0_/RC), following Strasser et al. (2004).

Leaf spectral reflectance was assessed to characterize the optical and physiological responses of plants to NaCl and PGPB treatments. Measurements were performed using a PolyPen RP 410 (NIR) spectrometer (Photon Systems Instruments, Brno, Czech Republic), capturing reflectance spectra in the 627–1060 nm range. A suite of reflectance indices were calculated from the measured leaf spectra, including the normalized difference vegetation index: NDVI = (RNIR—Rred) / (RNIR + Rred) (Rouse et al. 1974), greenness index: G = R554 / R677 (Zarco-Tejada et al., 2005), modified chlorophyll absorption ratio index: MCARI = [(R700 – R670) – 0.2 * (R700 – R550)] * (R700/ R670) (Daughtry et al. 2000), modified chlorophyll absorption ratio index 1: MCARI1 = 1.2 * [2.5 * (R790 – R670) – 1.3 * (R790 – R550)] (Haboudane et al. 2004), transformed chlorophyll absorption ratio index: TCARI = 3 * [(R700 – R670) – 0.2 * (R700 – R550) * (R700 / R670)] (Haboudane et al. 2002), Carotenoid Reflectance Index 1: CRI1 = 1 / R510 – 1 / R550 (Gitelson et al. 2002), Carotenoid Reflectance Index 2: CRI2 = 1 / R510 – 1 / R700 (Gitelson et al. 2002), structure insensitive pigment index: SIPI = (R790 – R450) / (R790 – R650) (Peñuelas et al. 2002). Normalized Phaeophytinization Index: NPQI = (R415 – R435) / (R415 + R435) (Peñuelas et al. 2002), and photochemical reflectance index: PRI = (R570 – R531) / (R570 + R531) (Gamon et al. 1997).

### Chlorophyll and carotenoid content determination

Chlorophyll a, chlorophyll b, total chlorophyll, and carotenoid contents were determined using a refined spectrophotometric method based on Wellburn (1994). Fresh leaf tissue (0.5 g) of *N*. *tabacum* was homogenized in 10 mL of 80% (v/v) acetone under dim light to minimize pigment degradation. The homogenate was centrifuged at 5,000 × g for 10 min at 4 °C, and the supernatant was collected for analysis. Absorbance was measured at 663.2 nm, 646.8 nm, and 470 nm using a UV–Visible spectrophotometer (Specord 205, Analytik Jena, Germany). Pigment concentrations (mg/g FW) were calculated using the following equations:

Chlorophyll a (mg/g FW) = (12.25 × A663.2 – 2.79 × A646.8) × V / (1000 × W).

Chlorophyll b (mg/g FW) = (21.50 × A646.8 – 5.10 × A663.2) × V / (1000 × W).

Total Chlorophyll (mg/g FW) = (7.15 × A663.2 + 18.71 × A646.8) × V / (1000 × W).

Carotenoids (mg/g FW) = [(1000 × A470) – (1.82 × Chlorophyll a) – (85.02 × Chlorophyll b)] / 198 × V / (1000 × W).

### Antioxidant enzyme analysis (SOD, POD, CAT, and APX)

To assess the antioxidant defense response, the activities of superoxide dismutase (SOD), peroxidase (POD), catalase (CAT), and ascorbate peroxidase (APX) were determined in leaf tissues of *N*. *tabacum*. Fresh leaf tissue (0.1 g) from each treatment group was homogenized in 1 mL of ice-cold 50 mM potassium phosphate buffer (pH 7.4) containing 0.5 mM EDTA, using a pre-chilled mortar and pestle. The homogenates were centrifuged at 15,000 × g for 15 min at 4 °C, and the resulting supernatants were collected and kept on ice for subsequent enzyme assays.

SOD activity was measured according to the method of Beauchamp and Fridovich (1971), based on the inhibition of nitroblue tetrazolium (NBT) photoreduction. The 3 mL reaction mixture contained 50 mM phosphate buffer (pH 7.8), 75 µM NBT, 0.1 mM EDTA, 2 µM riboflavin, 13 mM methionine, and 100 µL of enzyme extract. The mixture was illuminated for 30 min using cool white fluorescent tubes at an irradiance of approximately 120 µmol photons m⁻^2^ s⁻^1^, and absorbance was recorded at 560 nm using a UV–Vis spectrophotometer (Specord 205, Analytik Jena, Germany). One unit of SOD activity was defined as the amount of enzyme required to inhibit NBT reduction by 50% per minute per mg of protein.

POD activity was assayed following the guaiacol oxidation method described by Castillo et al. (1984). The 3 mL reaction mixture consisted of 100 mM potassium phosphate buffer (pH 6.1), 20 mM guaiacol, 12 mM H₂O₂, and 100 µL of enzyme extract. The increase in absorbance at 470 nm, due to tetraguaiacol formation, was monitored for 1 min. POD activity was calculated using an extinction coefficient of 25.5 mM⁻^1^ cm⁻^1^ and expressed as µmol of tetraguaiacol formed per minute per mg of protein.

CAT activity was determined by measuring the decomposition of hydrogen peroxide at 240 nm, following the method of Aebi (1974). The 3 mL reaction mixture included 100 mM potassium phosphate buffer (pH 7.0), 75 mM H₂O₂, and 100 µL of enzyme extract. The decrease in absorbance at 240 nm was recorded for 1 min, and CAT activity was calculated using an extinction coefficient of 39.4 mM⁻^1^ cm⁻^1^. One unit of CAT activity was defined as the amount of enzyme required to decompose 1 µmol of H₂O₂ per minute per mg of protein.

APX activity was determined based on the method of Nakano and Asada (1981), by monitoring the oxidation of ascorbate at 290 nm. The 1.8 mL reaction mixture contained 50 mM potassium phosphate buffer (pH 7.0), 0.1 mM ascorbate, 0.12 mM H₂O₂, and 100 µL of enzyme extract. The decrease in absorbance at 290 nm was recorded for 1 min, and APX activity was calculated using an extinction coefficient of 2.8 mM⁻^1^ cm⁻^1^. Results were expressed as µmol of ascorbate oxidized per minute per mg of protein.

### Statistical analysis

All experiments were conducted with five independent biological replicates per treatment, following a completely randomized design (CRD). Data are presented as mean ± standard deviation (SD). A two-way analysis of variance (ANOVA) was performed using IBM SPSS Statistics v30 to assess the individual and interactive effects of bacterial inoculation and salinity stress. Two-way ANOVA was used to test the main effects of salinity level and bacterial inoculation, as well as their interaction (salinity × inoculation), followed by Tukey’s HSD post hoc test at a 5% significance level (p < 0.05). Graphs were generated in GraphPad Prism v10.1.2. Hierarchical clustered heatmaps, Pearson’s correlation analyses, and OJIP fluorescence curve analysis were performed in Python v3.11, using Pandas (v2.2), Seaborn (v0.12.2), and Matplotlib (v3.8) for data handling and visualization. Principal component analysis (PCA) was conducted using OriginPro 2024 (v10.1.0.178).

## Results

### Bacterial growth and salt tolerance

**Fig. 1:**
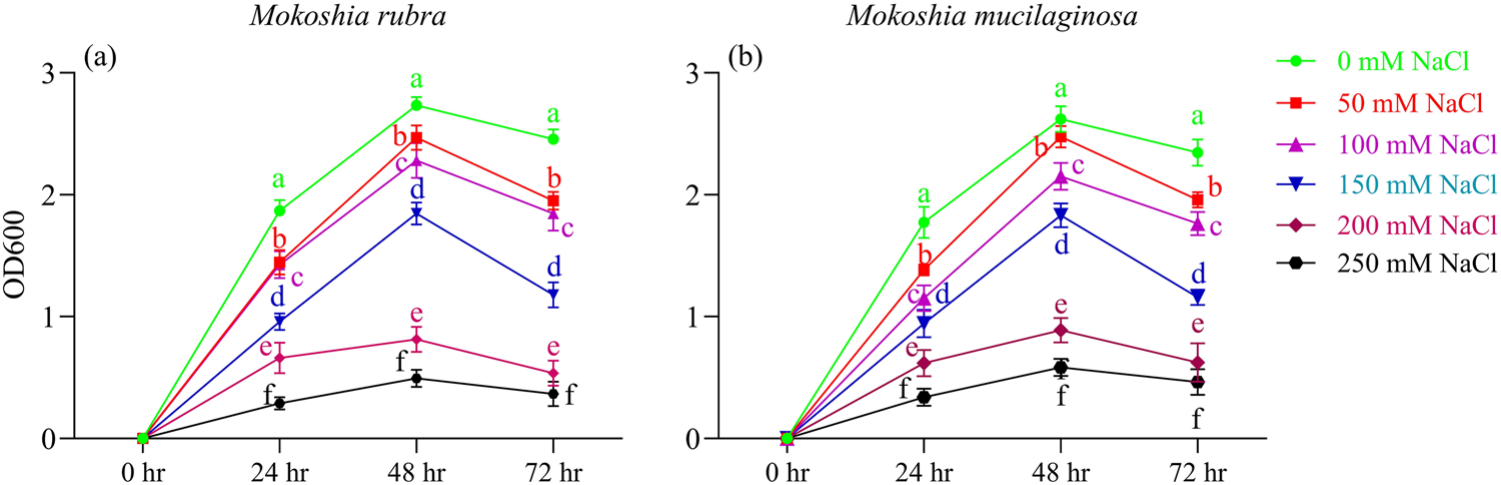
Growth of (**a**) *M*. *rubra* and (**b**) *M*. *mucilaginosa* assessed by optical density at 600 nm (OD₆₀₀) after 0, 24, 48, and 72 h under varying NaCl concentrations (0, 50, 100, 150, 200, and 250 mM). Values represent mean ± SD (n = 5). Different lowercase letters at each time point indicate significant differences among treatments (two-way ANOVA, Tukey’s HSD, p < 0.05)

The growth of *M*. *rubra* and *M*. *mucilaginosa* under varying NaCl concentrations was monitored over a 72 h period (Fig. 1a, b). Both strains followed a typical growth pattern, with OD₆₀₀ values increasing up to 48 h and decreasing at 72 h. In control conditions (0 mM NaCl), both species exhibited the highest growth, reaching peak OD₆₀₀ at 48 h. Increasing NaCl concentrations progressively suppressed bacterial proliferation. At moderate salt levels (50–150 mM), growth was reduced compared to the control but remained substantial at 48 h. However, at 200 mM and especially at 250 mM NaCl, growth was significantly impaired across all time points (P < 0.05), as reflected by markedly lower OD₆₀₀ values. The results indicate that both *M*. *rubra* and *M*. *mucilaginosa* exhibit tolerance to NaCl stress up to 150 mM, beyond which growth is significantly inhibited, suggesting a threshold of salt tolerance for these strains.

To further evaluate the impact of *Mokoshia* inoculation under saline conditions, a series of plant-based experiments was conducted using *N*. *tabacum*. These experiments examined plant growth, photosynthetic performance, pigment stability, and antioxidant responses under increasing NaCl concentrations. The results of these analyses are presented below.

### Biomass accumulation

**Fig. 2:**
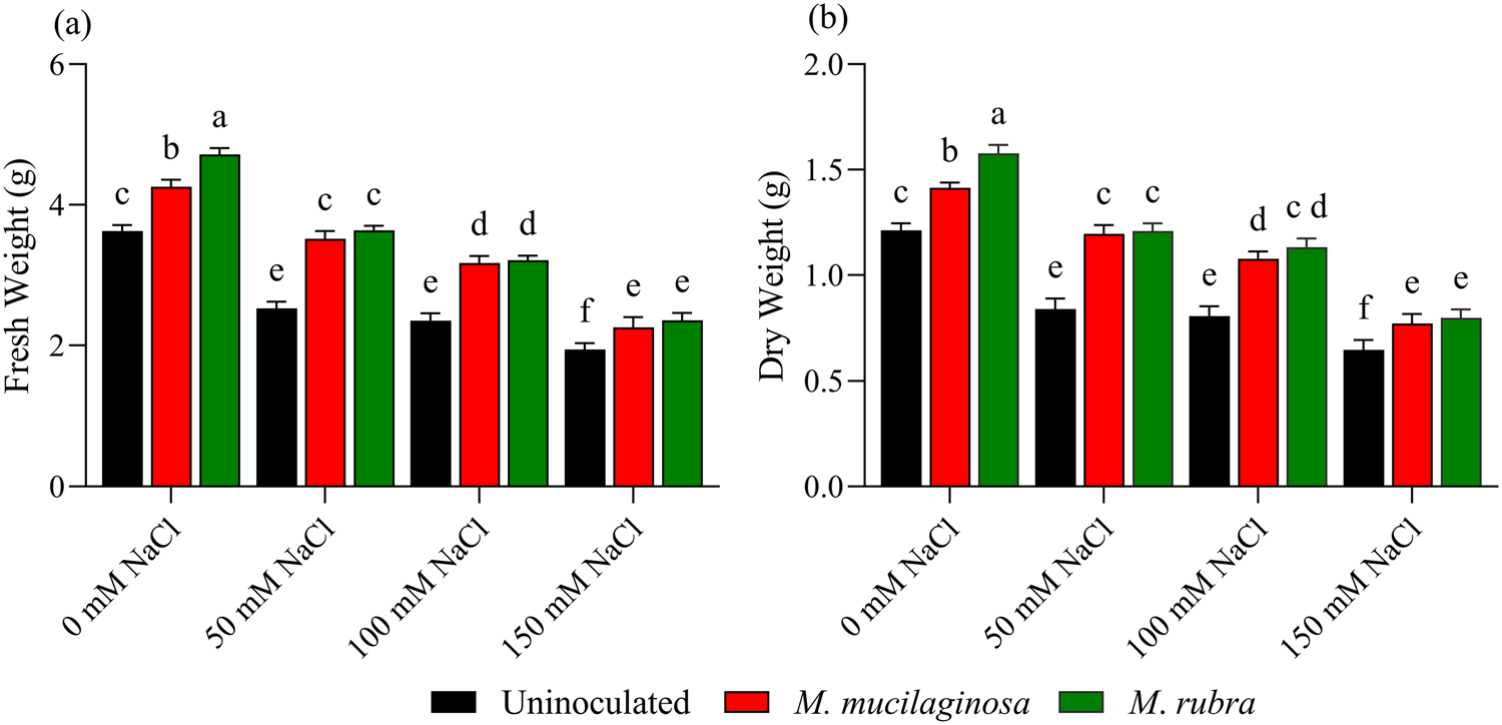
Effects of NaCl stress and bacterial inoculation on plant biomass. **a** Fresh weight and **b** dry weight of *N*. *tabacum* treated with 0, 50, 100, and 150 mM NaCl, either uninoculated (black bars) or inoculated with *M*. *mucilaginosa* (red bars) or *M*. *rubra* (green bars). Values are mean ± SD (n = 5). Different lowercase letters indicate significant differences among treatments (two-way ANOVA, Tukey’s HSD, p < 0.05)

Salinity stress markedly reduced the growth of N. tabacum, as evidenced by declines in both fresh and dry biomass (Fig. 2a, b). In uninoculated plants, fresh weight (FW) decreased by 1.43-, 1.54-, and 1.87-fold under 50, 100, and 150 mM NaCl, respectively, relative to the non-stressed control (p < 0.05). Inoculation with *M*. *mucilaginosa* and *M*. *rubra* partially alleviated this reduction, with higher FW values observed in inoculated plants across salinity levels. Under 150 mM NaCl, FW increased by 1.39- and 1.44-fold in plants inoculated with *M*. *mucilaginosa* and *M*. *rubra*, respectively, compared with uninoculated plants (p < 0.05). Similarly, dry weight (DW) in uninoculated plants declined by 1.44-, 1.50-, and 1.88-fold under 50, 100, and 150 mM NaCl treatments, respectively (p < 0.05). Inoculated plants showed higher DW values under salinity stress; at 150 mM NaCl, *M*. *mucilaginosa* and *M*. *rubra* increased DW by 1.19- and 1.23-fold, respectively, relative to uninoculated plants (p < 0.05). Overall, both bacterial strains significantly promoted plant growth under non-saline conditions, while under increasing salinity their growth-promoting effects were reduced but remained evident, particularly at moderate NaCl levels. Although *M*. *rubra* often showed slightly higher mean values than *M*. *mucilaginosa*, differences between strains were not statistically significant under saline conditions. Two-way ANOVA revealed significant main effects of salinity and bacterial inoculation on fresh and dry biomass, as well as a significant salinity × inoculation interaction, indicating that the magnitude of bacterial growth promotion depended on salinity level, with diminishing effects on biomass at higher NaCl concentrations.

### Chlorophyll fluorescence and PSII efficiency

**Fig. 3:**
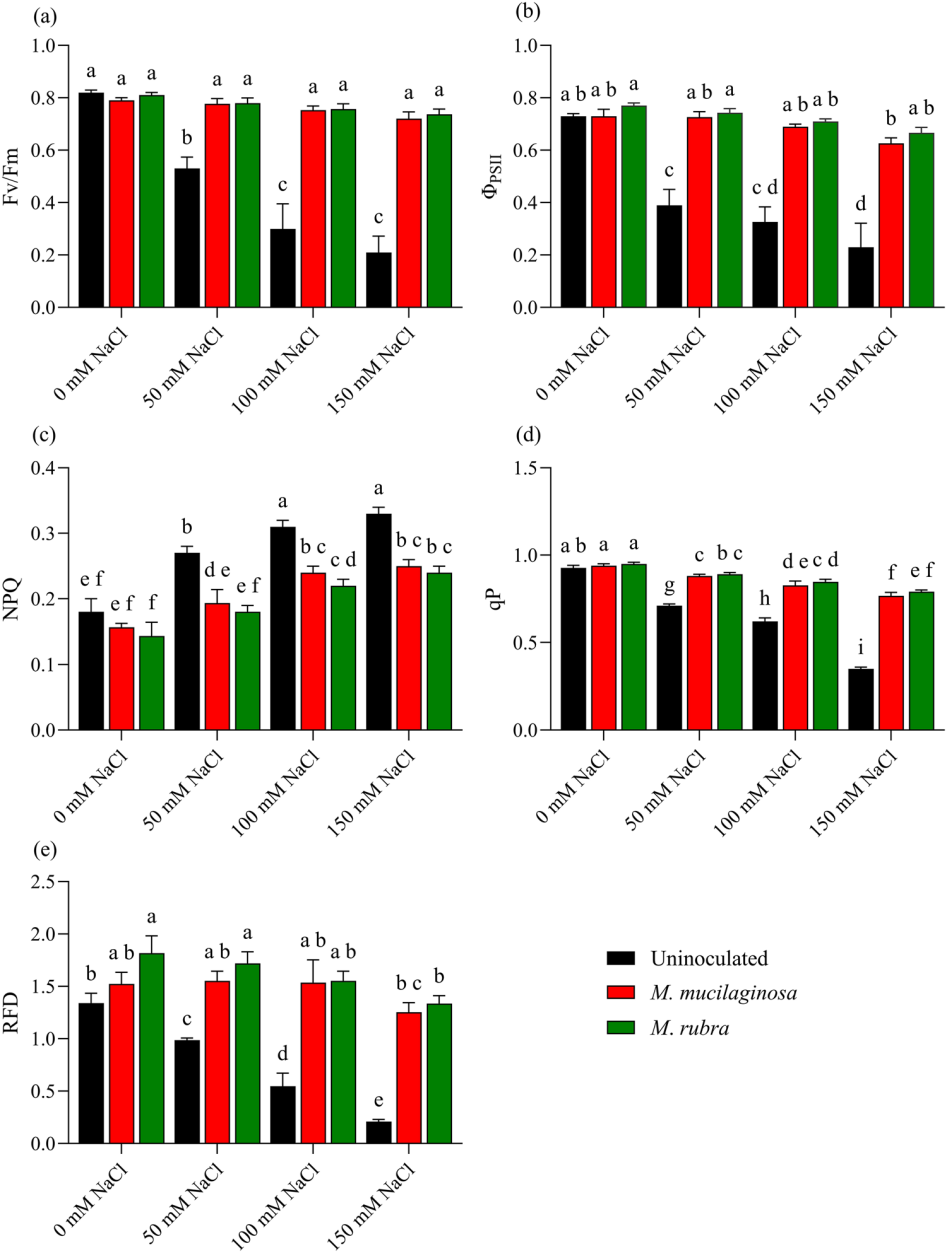
Photosynthetic performance of *N*. *tabacum* under NaCl stress and bacterial inoculation. Chlorophyll fluorescence parameters were measured in plants treated with 0, 50, 100, and 150 mM NaCl, either uninoculated (black bars) or inoculated with *M*. *mucilaginosa* (red bars) or *M*. *rubra* (green bars). Parameters: **a** Fv/Fm, **b** Φ_PSII_, **c** NPQ, **d** qP, and **e** RFD. Values are mean ± SD (n = 5). Different lowercase letters indicate significant differences among treatments (two-way ANOVA, Tukey’s HSD, p < 0.05)

Salinity stress impaired the photochemical performance of *N*. *tabacum*, as reflected by changes in key chlorophyll fluorescence parameters. In uninoculated plants, maximum photosynthetic efficiency (Fv/Fm) declined by 1.35-, 2.54-, and 2.55-fold under 50, 100, and 150 mM NaCl, respectively, relative to the non-stressed control (p < 0.05; Fig. 3a). Inoculation with *M*. *mucilaginosa* and *M*. *rubra* attenuated this decline, with higher Fv/Fm values observed in inoculated plants across salinity levels. At 150 mM NaCl, Fv/Fm increased by 2.47- and 2.51-fold, respectively, compared with uninoculated plants (p < 0.05). A similar pattern was observed for effective quantum yield (Φ_PSII_), which decreased by 1.34-, 2.16-, and 2.50-fold under increasing NaCl concentrations in uninoculated plants (p < 0.05; Fig. 3b). Inoculation with *M*. *mucilaginosa* and *M*. *rubra* partially reversed this reduction, with ΦPSII increasing by 1.99- and 2.29-fold, respectively, at 150 mM NaCl (p < 0.05). Non-photochemical quenching (NPQ) increased in uninoculated plants by 1.47-, 1.78-, and 2.01-fold under 50, 100, and 150 mM NaCl, respectively (p < 0.05; Fig. 3c). In contrast, inoculated plants showed lower NPQ values under salinity stress, with reductions of 1.63- and 1.66-fold observed for *M*. *mucilaginosa* and *M*. *rubra*, respectively, at 150 mM NaCl (p < 0.05). Photochemical quenching (qP) declined by 1.30-, 1.53-, and 1.77-fold in uninoculated plants under increasing salinity (p < 0.05; Fig. 3d), whereas inoculated plants exhibited higher qP values, with 2.06- and 2.12-fold increases recorded for *M*. *mucilaginosa* and *M*. *rubra*, respectively, at 150 mM NaCl (p < 0.05). Similarly, Relative fluorescence decrease (RFD) decreased by 1.74-, 2.65-, and 3.34-fold under 50, 100, and 150 mM NaCl in uninoculated plants (p < 0.05; Fig. 3e). Inoculation mitigated this decline, with RFD increasing by 2.55- and 2.87-fold in *M*. *mucilaginosa*- and *M*. *rubra*-treated plants, respectively, at 150 mM NaCl compared with uninoculated controls (p < 0.05). For photosynthetic parameters, two-way ANOVA revealed significant main effects of salinity and bacterial inoculation, as well as a significant salinity × inoculation interaction. These results indicate that the magnitude of bacterial effects on photochemical performance depended on NaCl concentration. Chlorophyll fluorescence imaging was used to qualitatively visualize spatial patterns of photosynthetic performance across leaf surfaces (Supplementary Figures S1–S5). As leaf size varied due to salinity-induced growth effects, imaging was employed as a supportive visualization tool rather than for quantitative comparison.

### OJIP transients

**Fig. 4:**
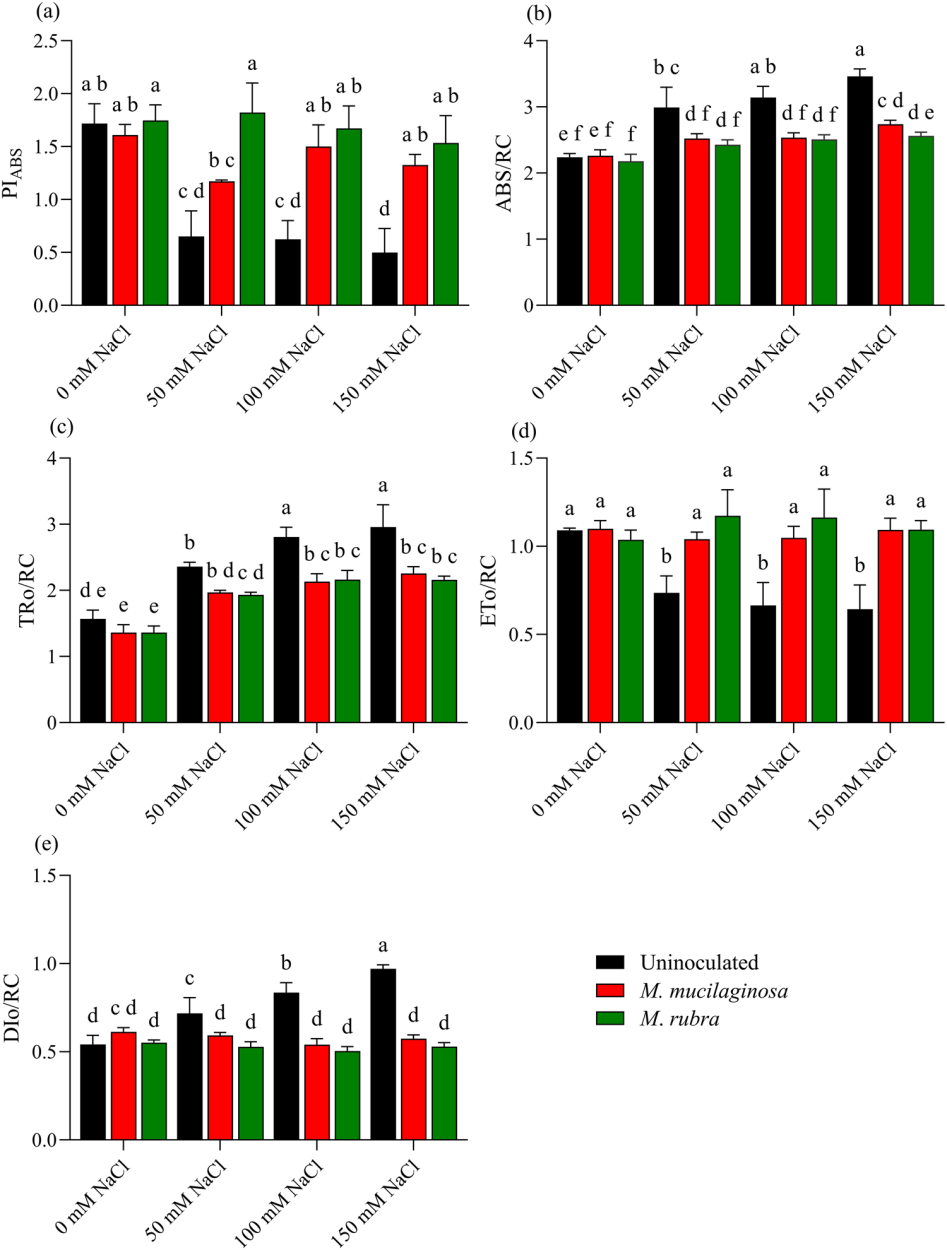
PSII energy flux parameters in *N*. *tabacum* under NaCl stress and bacterial inoculation. Plants were treated with 0, 50, 100, and 150 mM NaCl, either uninoculated (black bars) or inoculated with *M*. *mucilaginosa* (red bars) or *M*. *rubra* (green bars). Parameters: **a** PI_ABS_, **b** ABS/RC, **c **TRo/RC, **d** ETo/RC, **e** DIo/RC. Values are mean ± SD (n = 5). Different lowercase letters indicate significant differences among treatments (two-way ANOVA, Tukey’s HSD, p < 0.05)

Salinity stress altered the photosynthetic performance of *N*. *tabacum*, as reflected by changes in OJIP fluorescence parameters. The performance index on absorption basis (PI_ABS_) declined markedly in uninoculated plants under NaCl stress. Relative to the non-stressed control, PI_ABS_ decreased by 2.0-, 2.75-, and 3.45-fold under 50, 100, and 150 mM NaCl, respectively (p < 0.05; Fig. 4a). Inoculation with *M*. *mucilaginosa* or *M*. *rubra* attenuated this decline, with PI_ABS_ increasing by 2.66- and 3.08-fold, respectively, at 150 mM NaCl compared with uninoculated plants (p < 0.05). Absorbed energy flux per reaction center (ABS/RC) increased progressively with salinity in uninoculated plants, showing 1.27-, 1.33-, and 1.55-fold increases at 50, 100, and 150 mM NaCl, respectively (p < 0.05; Fig. 4b). Inoculated plants exhibited lower ABS/RC values under salinity stress, with both bacterial treatments differing significantly from uninoculated stressed plants (p < 0.05). Similarly, trapped energy flux per reaction center (TRo/RC) increased by 1.38-, 1.78-, and 2.06-fold in uninoculated plants under increasing NaCl concentrations (p < 0.05; Fig. 4c). Inoculation moderated this increase, with TRo/RC values remaining lower than those observed in uninoculated plants at corresponding salinity levels (p < 0.05). Electron transport flux per reaction center (ETo/RC) declined by 1.28-, 1.57-, and 1.64-fold at 50, 100, and 150 mM NaCl, respectively, in uninoculated plants (p < 0.05; Fig. 4d). Both bacterial strains increased ETo/RC relative to uninoculated plants under salinity stress, with approximately 1.70-fold higher values observed at 150 mM NaCl (p < 0.05). In contrast, dissipated energy flux per reaction center (DIo/RC) increased by 1.16-, 1.51-, and 1.79-fold in uninoculated plants under 50, 100, and 150 mM NaCl, respectively (p < 0.05; Fig. 4e). Inoculation reduced DIo/RC values under salinity stress, with 1.69- and 1.83-fold decreases observed for *M*. *mucilaginosa* and *M*. *rubra*, respectively, at 150 mM NaCl compared with uninoculated plants (p < 0.05). Two-way ANOVA followed by Tukey’s post hoc test revealed significant main effects of salinity and bacterial inoculation, as well as significant salinity × inoculation interactions for OJIP parameters (p < 0.05). Normalized OJIP fluorescence transients were analyzed to further examine stress-induced alterations in PSII energy fluxes; detailed descriptions and corresponding figures are provided in the Supplementary Materials (Fig. S6). L- and K-band analyses were conducted to explore donor- and acceptor-side perturbations of PSII, with full descriptions and figures presented in the Supplementary Materials (Fig. S7).

### Spectral indicators of leaf vitality

**Fig. 5:**
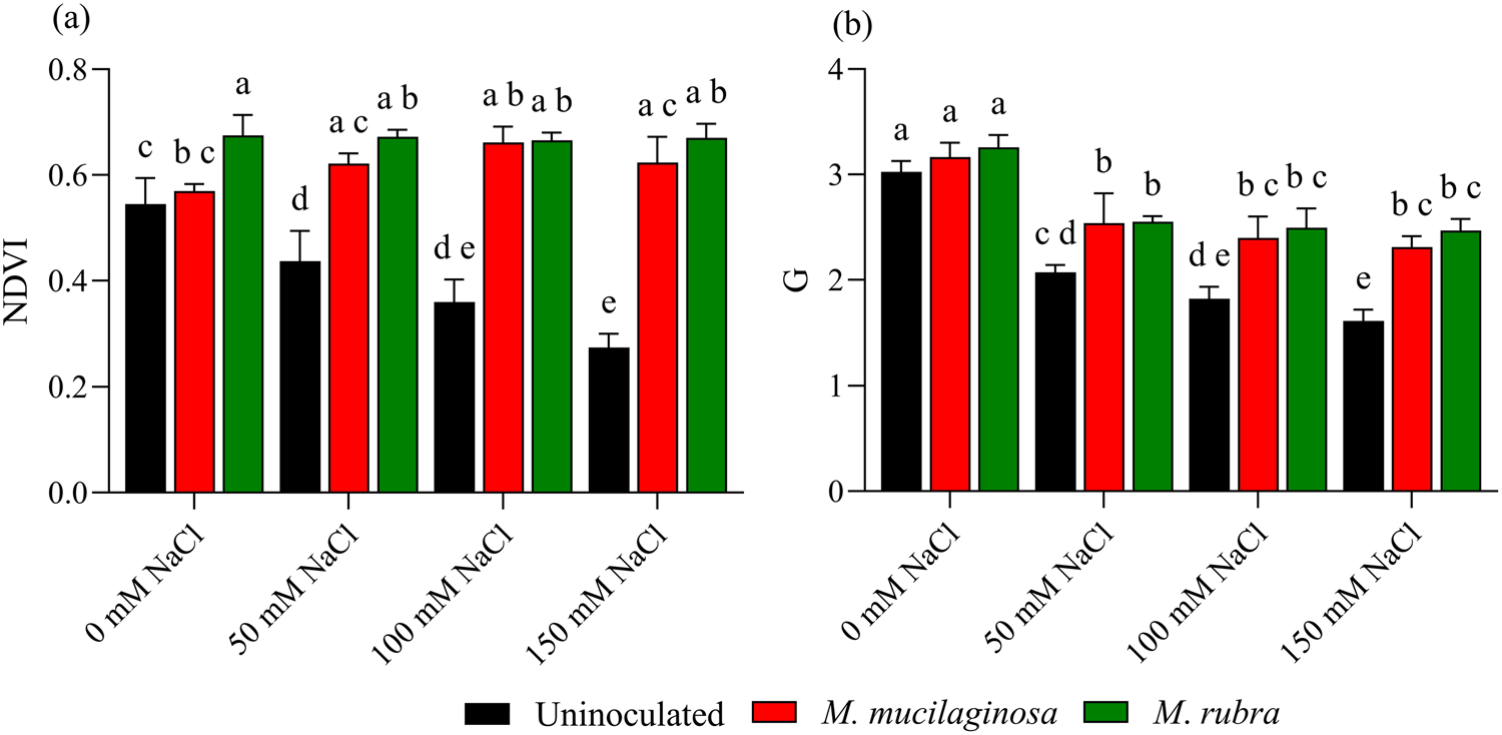
Vegetation indices in *N*. *tabacum* under NaCl stress and bacterial inoculation. **a** NDVI and **b** Greenness Index (G) were measured in plants treated with 0, 50, 100, and 150 mM NaCl, either uninoculated (black bars) or inoculated with *M*. *mucilaginosa* (red bars) or *M*. *rubra* (green bars). Values are mean ± SD (n = 5). Different lowercase letters indicate significant differences among treatments (two-way ANOVA, Tukey’s HSD, p < 0.05)

Salinity stress affected vegetative reflectance traits of *N*. *tabacum*, as reflected by changes in the normalized difference vegetation index (NDVI) and greenness index (G) (Fig. 5a, b). In uninoculated plants, NDVI declined by 1.24-, 1.49-, and 1.98-fold under 50, 100, and 150 mM NaCl, respectively, relative to the non-stressed control (p < 0.05; Fig. 5a). Inoculated plants exhibited higher NDVI values under salinity stress. At 150 mM NaCl, NDVI increased by 2.20- and 2.34-fold in plants treated with *M*. *mucilaginosa* and *M*. *rubra*, respectively, compared with uninoculated plants (p < 0.05). Similarly, the greenness index (G) declined in uninoculated plants by 1.44-, 1.62-, and 1.79-fold under 50, 100, and 150 mM NaCl, respectively (p < 0.05; Fig. 5b). Inoculation mitigated this decline, with G values increasing by 1.34- and 1.39-fold in *M*. *mucilaginosa*- and *M*. *rubra*-treated plants, respectively, at 150 mM NaCl relative to uninoculated stressed plants (p < 0.05). Two-way ANOVA followed by Tukey’s post hoc test revealed significant main effects of salinity and bacterial inoculation on NDVI and G values, as well as significant salinity × inoculation interactions (p < 0.05).

### Chlorophyll-related spectral indices

**Fig. 6:**
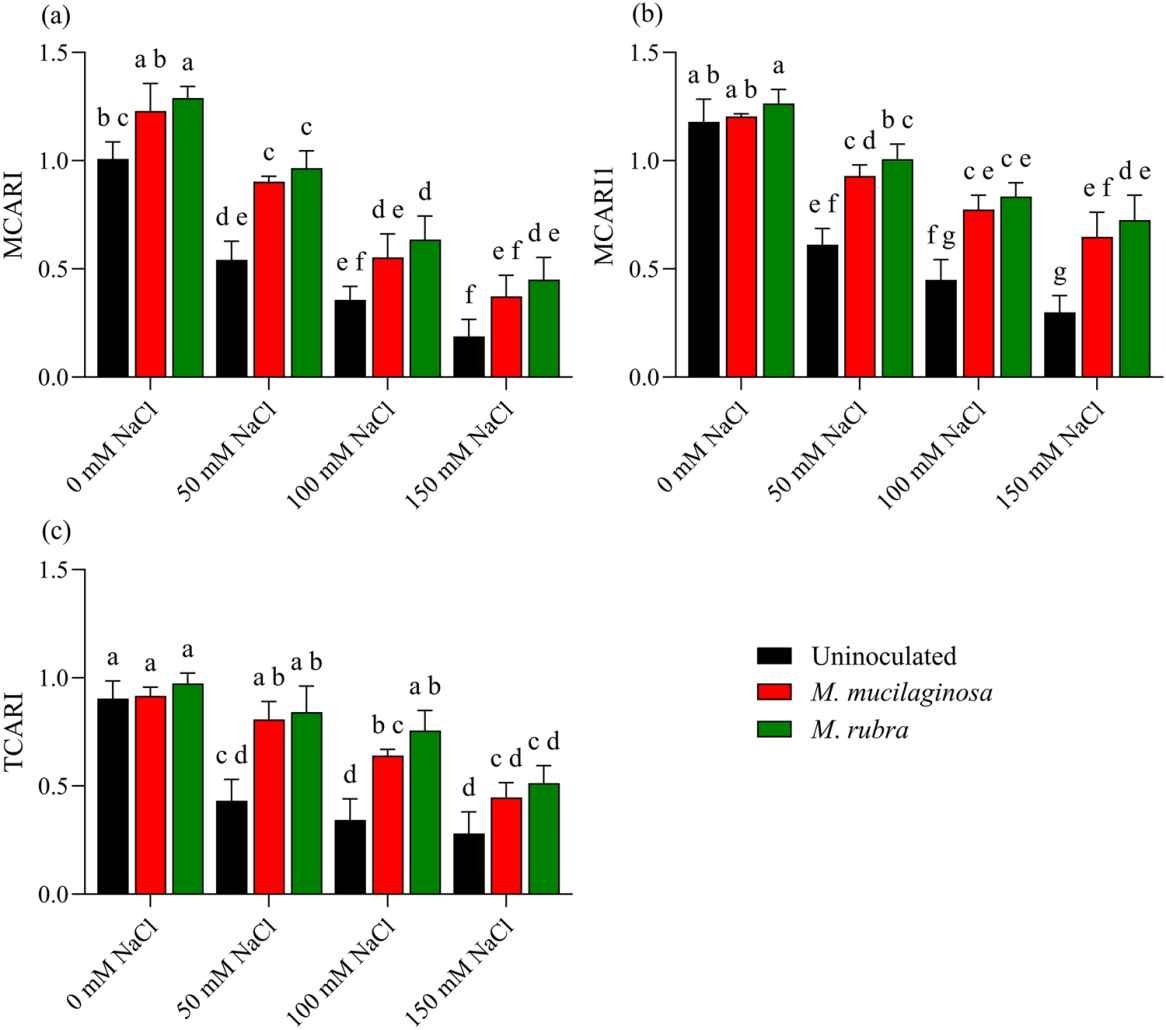
Reflectance-based chlorophyll indices in *N*. *tabacum* under NaCl stress and bacterial inoculation. **a** MCARI, **b** MCARI1, and **c** TCARI were measured in plants treated with 0, 50, 100, and 150 mM NaCl, either uninoculated (black bars) or inoculated with *M*. *mucilaginosa* (red bars) or *M*. *rubra* (green bars). Values are mean ± SD (n = 5). Different lowercase letters indicate significant differences among treatments (two-way ANOVA, Tukey’s HSD, p < 0.05)

Salinity stress affected chlorophyll-related reflectance indices of *N*. *tabacum*, as reflected by changes in MCARI, MCARI1, and TCARI (Fig. 6). In uninoculated plants, Modified Chlorophyll Absorption Ratio Index (MCARI) declined by 1.86-, 2.82-, and 5.34-fold under 50, 100, and 150 mM NaCl, respectively, relative to the non-stressed control (p < 0.05; Fig. 6a). Inoculated plants exhibited higher MCARI values under salinity stress. At 150 mM NaCl, MCARI increased by 1.98- and 2.39-fold in plants treated with *M*. *mucilaginosa* and *M*. *rubra*, respectively, compared with uninoculated plants (p < 0.05). A similar trend was observed for MCARI1, which declined by 1.93-, 2.63-, and 3.94-fold in uninoculated plants under increasing NaCl concentrations (p < 0.05; Fig. 6b). Inoculation mitigated this decline, with MCARI1 values increasing by 2.16- and 2.42-fold in *M*. *mucilaginosa*- and *M*. *rubra* -treated plants, respectively, at 150 mM NaCl relative to uninoculated controls (p < 0.05). The transformed chlorophyll absorption reflectance index (TCARI) was also reduced by 2.10-, 2.63-, and 3.23-fold in uninoculated plants under 50, 100, and 150 mM NaCl, respectively (p < 0.05; Fig. 6c). Inoculated plants showed higher TCARI values under salinity stress, with 1.59- and 1.83-fold increases observed for *M*. *mucilaginosa* and *M*. *rubra*, respectively, at 150 mM NaCl compared with uninoculated plants (p < 0.05). Two-way ANOVA followed by Tukey’s post hoc test revealed significant main effects of salinity and bacterial inoculation, as well as significant salinity × inoculation interactions for all chlorophyll-related spectral indices (p < 0.05).

### Pigment-related spectral indices

**Fig. 7:**
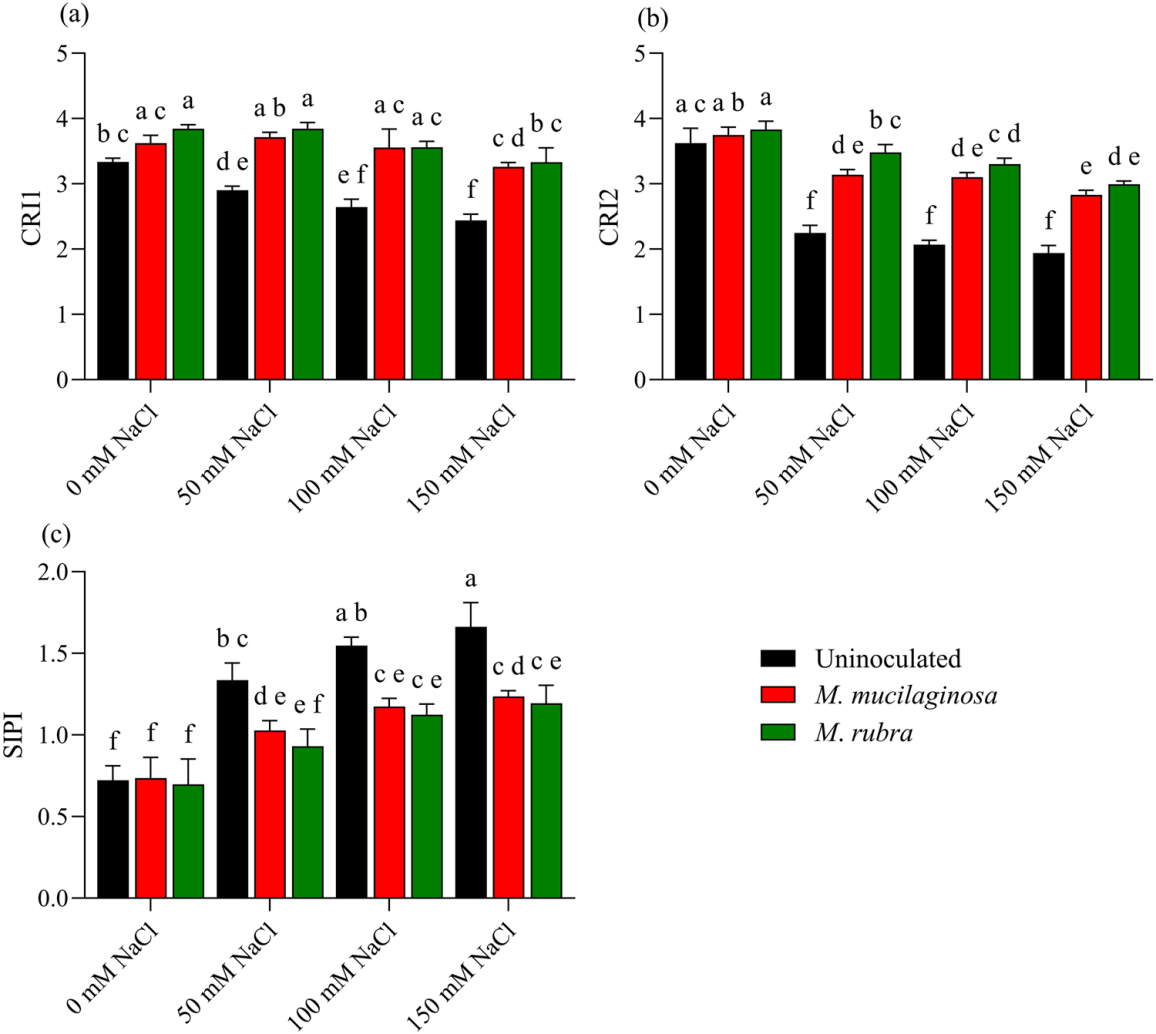
Spectral pigment indices reflecting carotenoid composition and pigment stability in *N*. *tabacum* under NaCl stress and bacterial inoculation. **a** CRI1, **b** CRI2, and **c** SIPI were measured in plants treated with 0, 50, 100, and 150 mM NaCl, either uninoculated (black bars) or inoculated with *M*. *mucilaginosa* (red bars) or *M*. *rubra* (green bars). Values are mean ± SD (n = 5). Different lowercase letters indicate significant differences among treatments (two-way ANOVA, Tukey’s HSD, p < 0.05)

Salinity stress affected carotenoid-related spectral indices of *N*. *tabacum*, as reflected by changes in CRI1, CRI2, and SIPI (Fig. 7a–c). In uninoculated plants, Carotenoid Reflectance Index 1 (CRI1) declined by 1.22-, 1.40-, and 1.53-fold under 50, 100, and 150 mM NaCl, respectively, relative to the non-stressed control (p < 0.05; Fig. 7a). Inoculated plants exhibited higher CRI1 values under salinity stress. At 150 mM NaCl, CRI1 increased by 1.48- and 1.57-fold in *M*. *mucilaginosa*- and *M*. *rubra*-treated plants, respectively, compared with uninoculated plants (p < 0.05). A similar pattern was observed for CRI2, which declined by 1.57-, 1.74-, and 1.90-fold in uninoculated plants under 50, 100, and 150 mM NaCl, respectively (p < 0.05; Fig. 7b). Inoculation mitigated this decline, with CRI2 values increasing by 1.48- and 1.57-fold in *M*. *mucilaginosa*- and *M*. *rubra*-treated plants, respectively, at 150 mM NaCl relative to uninoculated controls (p < 0.05). The structure insensitive pigment index (SIPI) increased in uninoculated plants by 1.38-, 2.06-, and 2.38-fold under 50, 100, and 150 mM NaCl, respectively (p < 0.05; Fig. 7c). Inoculated plants showed lower SIPI values under salinity stress, with reductions of 1.38- and 1.42-fold observed for *M*. *mucilaginosa* and *M*. *rubra*, respectively, at 150 mM NaCl compared with uninoculated plants (p < 0.05). Two-way ANOVA followed by Tukey’s post hoc test revealed significant main effects of salinity and bacterial inoculation, as well as significant salinity × inoculation interactions for all carotenoid-related spectral indices (p < 0.05).

### Pigment stress indices and photochemical reflectance

**Fig. 8:**
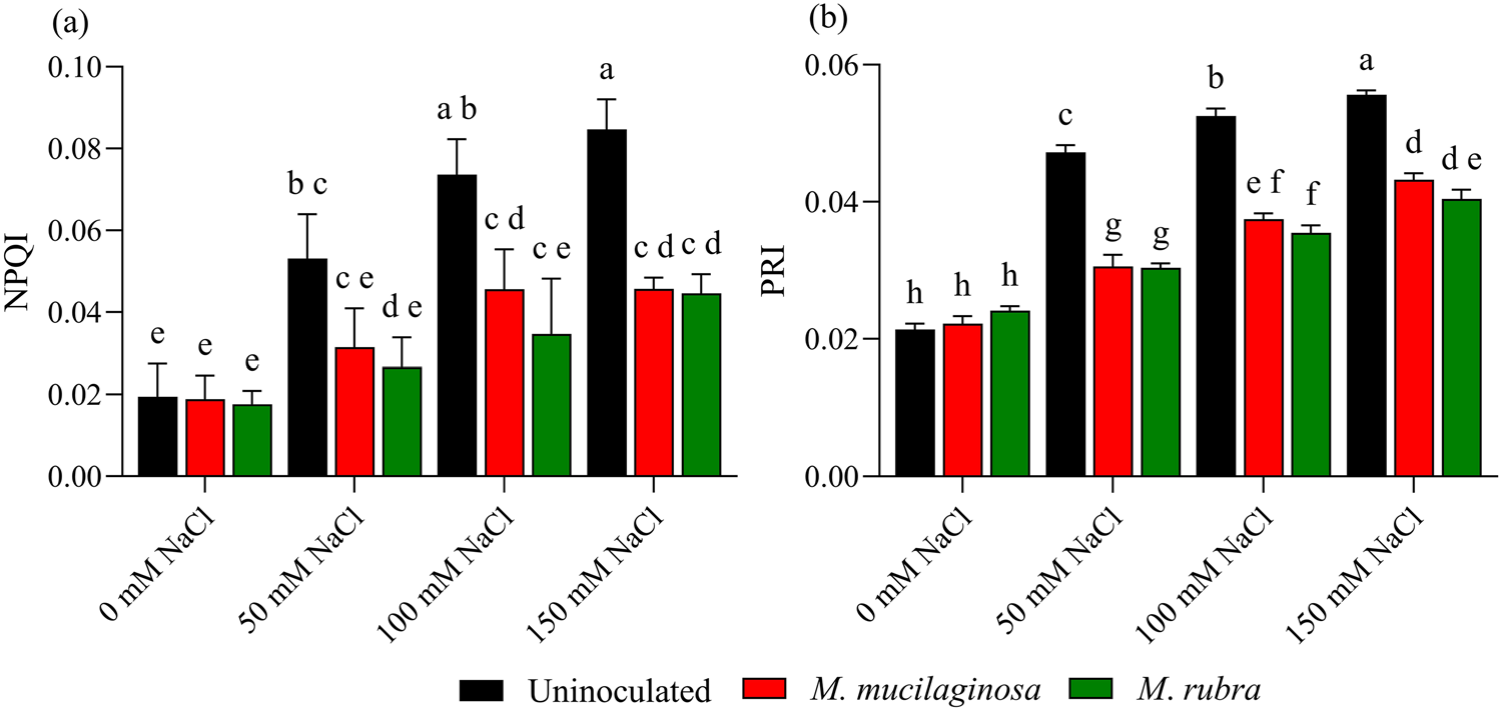
Pigment stress indices and photochemical reflectance in *N*. *tabacum* under NaCl stress and bacterial inoculation. **a** NPQI and **b** PRI were measured in plants treated with 0, 50, 100, and 150 mM NaCl, either uninoculated (black bars) or inoculated with *M*. *mucilaginosa* (red bars) or *M*. *rubra* (green bars). Values are mean ± SD (n = 5). Different lowercase letters indicate significant differences among treatments (two-way ANOVA, Tukey’s HSD, p < 0.05)

Salinity stress affected pigment-related stress indices and photochemical reflectance of *N*. *tabacum*, as reflected by changes in NPQI and PRI (Fig. 8a–b). In uninoculated plants, the normalized phaeophytinization index (NPQI) increased by 2.70-, 3.85-, and 4.30-fold under 50, 100, and 150 mM NaCl, respectively, relative to the non-stressed control (p < 0.05; Fig. 8a). Inoculated plants exhibited lower NPQI values under salinity stress. At 150 mM NaCl, NPQI decreased by 1.90- and 2.06-fold in *M*. *mucilaginosa*- and *M*. *rubra*-treated plants, respectively, compared with uninoculated stressed plants (p < 0.05). Conversely, the photochemical reflectance index (PRI) increased in uninoculated plants by 2.03-, 2.42-, and 2.63-fold under 50, 100, and 150 mM NaCl, respectively, relative to the control (p < 0.05; Fig. 8b). Inoculated treatments showed lower PRI values under salinity stress. At 150 mM NaCl, PRI was reduced by 1.31- and 1.38-fold in *M*. *mucilaginosa*- and *M*. *rubra*-treated plants, respectively, compared with uninoculated stressed plants (p < 0.05). Two-way ANOVA followed by Tukey’s post hoc test revealed significant main effects of salinity and bacterial inoculation, as well as significant salinity × inoculation interactions for NPQI and PRI (p < 0.05).

### Photosynthetic pigment analysis

**Fig. 9:**
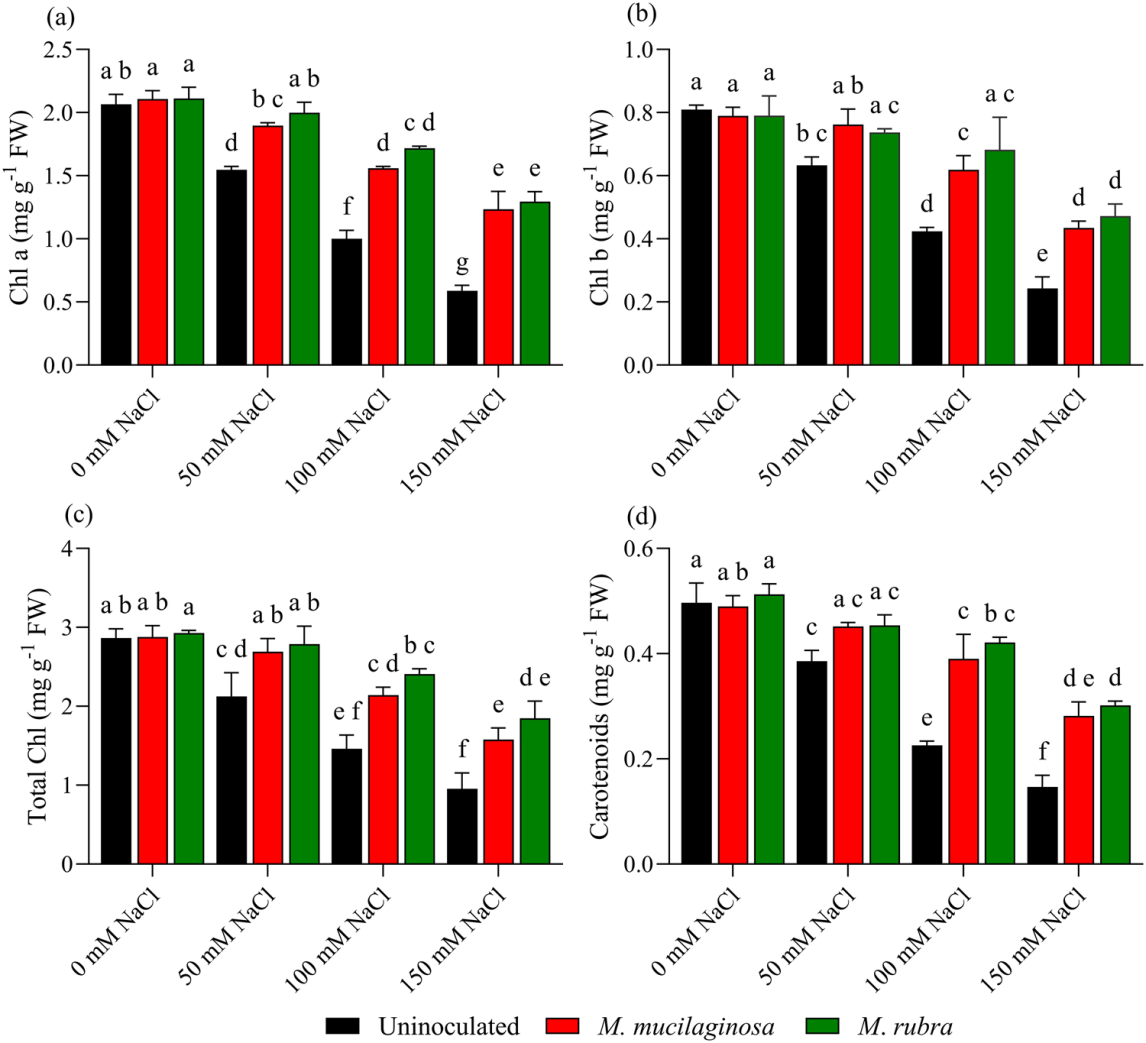
Effect of bacterial inoculation on chlorophyll and carotenoid contents in *N*. *tabacum* under salinity stress. **a** Chlorophyll a (Chl a), **b** Chlorophyll b (Chl b), **c** Total chlorophyll (T. Chl), and **d** Carotenoids were measured in plants treated with 0, 50, 100, and 150 mM NaCl, either uninoculated (black bars) or inoculated with *M*. *mucilaginosa* (red bars) or *M*. *rubra* (green bars). Values are mean ± SD (n = 5). Different lowercase letters indicate significant differences among treatments (two-way ANOVA, Tukey’s HSD, p < 0.05)

Salinity stress affected the photosynthetic pigment profile of *N*. *tabacum*, as reflected by changes in chlorophyll and carotenoid contents (Fig. 9a–d). In uninoculated plants, chlorophyll a (Chl a) declined by 1.33-, 2.06-, and 3.51-fold under 50, 100, and 150 mM NaCl, respectively, relative to the non-stressed control (p < 0.05; Fig. 9a). Inoculated plants exhibited higher Chl a levels under salinity stress. At 150 mM NaCl, Chl a increased by 2.10- and 2.20-fold in *M*. *mucilaginosa*- and *M*. *rubra*-treated plants, respectively, compared with uninoculated plants (p < 0.05). A similar trend was observed for chlorophyll b (Chl b), which decreased by 1.28-, 1.91-, and 3.33-fold in uninoculated plants under 50, 100, and 150 mM NaCl, respectively (p < 0.05; Fig. 9b). Inoculation mitigated this decline, with Chl b increasing by 1.79- and 1.94-fold in *M*. *mucilaginosa*- and *M*. *rubra*-treated plants, respectively, at 150 mM NaCl relative to uninoculated stressed plants (p < 0.05). Total chlorophyll content declined by 1.35-, 1.96-, and 3.01-fold in uninoculated plants under increasing NaCl concentrations (p < 0.05; Fig. 9c). Inoculated plants showed higher total chlorophyll levels under salinity stress, with 1.66- and 1.94-fold increases observed for *M*. *mucilaginosa* and *M*. *rubra*, respectively, at 150 mM NaCl compared with uninoculated plants (p < 0.05). Carotenoid content also declined under salinity stress, with 1.29-, 2.20-, and 3.38-fold reductions observed in uninoculated plants at 50, 100, and 150 mM NaCl, respectively (p < 0.05; Fig. 9d). Inoculation increased carotenoid levels under salinity stress, with 1.92- and 2.05-fold increases recorded for *M*. *mucilaginosa* and *M*. *rubra*, respectively, at 150 mM NaCl relative to uninoculated plants (p < 0.05). Two-way ANOVA followed by Tukey’s post hoc test revealed significant main effects of salinity and bacterial inoculation, as well as significant salinity × inoculation interactions for all pigment-related parameters (p < 0.05).

### Antioxidant enzyme activities

**Fig. 10:**
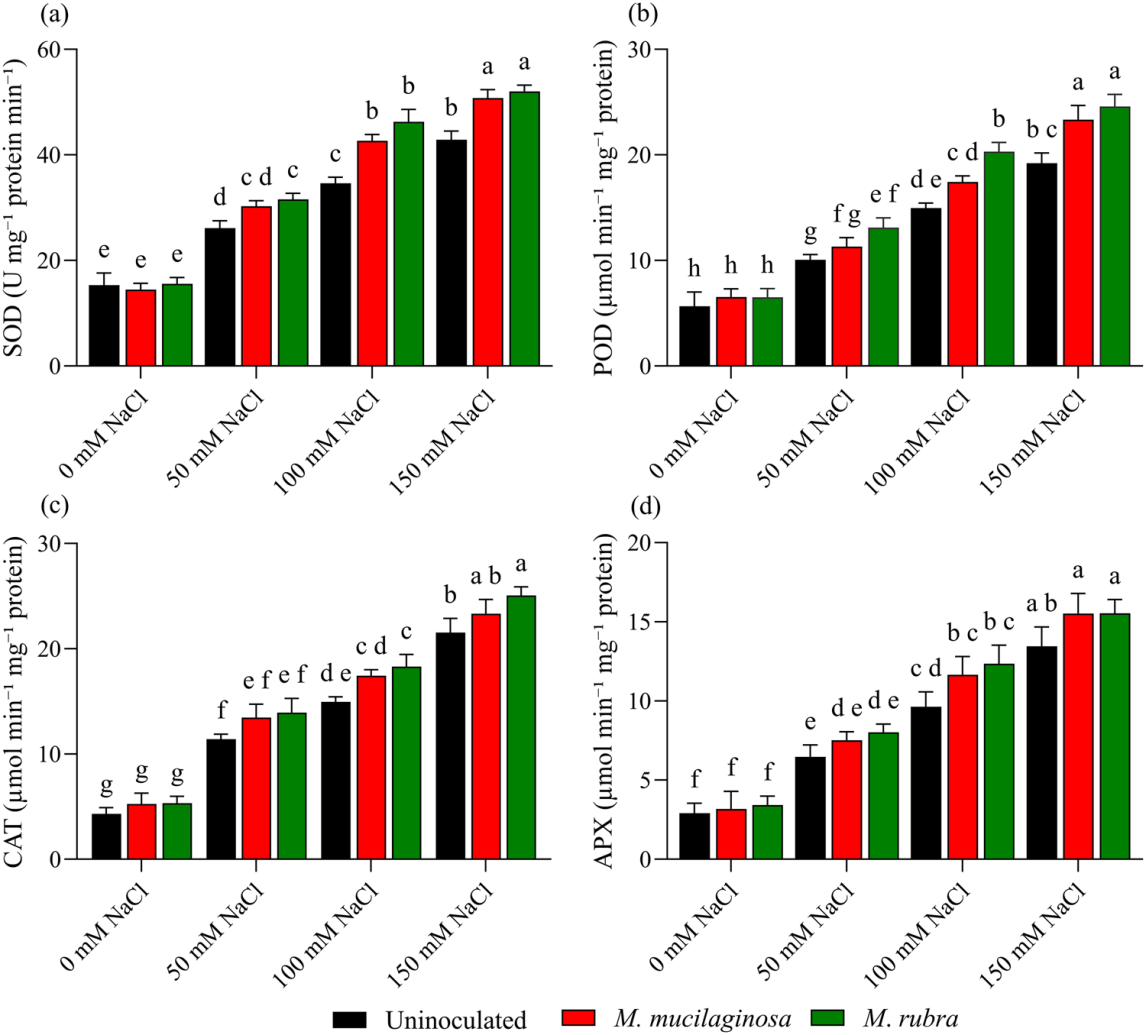
Antioxidant enzyme activity in *N*. *tabacum* leaves under salinity stress and bacterial inoculation. **a** Superoxide dismutase (SOD), **b** Peroxidase (POD), **c **Catalase (CAT), and **d** Ascorbate peroxidase (APX) were measured in plants treated with 0, 50, 100, and 150 mM NaCl, either uninoculated (black bars) or inoculated with *M*. *mucilaginosa* (red bars) or *M*. *rubra* (green bars). Values are mean ± SD (n = 5). Different lowercase letters indicate significant differences among treatments (two-way ANOVA, Tukey’s HSD, p < 0.05)

Salinity stress was associated with increased antioxidant enzyme activities in *N*. *tabacum*, as reflected by progressive increases in superoxide dismutase (SOD), peroxidase (POD), catalase (CAT), and ascorbate peroxidase (APX) with increasing NaCl concentrations in uninoculated plants compared with the non-saline control (Fig. 10a–d; p < 0.05). In uninoculated plants, SOD activity increased by 1.71-, 2.26-, and 2.80-fold at 50, 100, and 150 mM NaCl, respectively (Fig. 10a). Similarly, POD activity increased by 1.78-, 2.64-, and 3.39-fold (Fig. 10b), CAT activity by 2.39-, 2.76-, and 3.14-fold (Fig. 10c), and APX activity by 1.59-, 1.89-, and 2.23-fold (Fig. 10d) relative to the 0 mM NaCl control. Inoculation with *M*. *mucilaginosa* and *M*. *rubra* influenced antioxidant enzyme activities under salinity stress in an enzyme- and concentration-dependent manner. For SOD, both inoculants increased activity relative to uninoculated salt-stressed plants, with enhancements ranging from 1.16–1.26-fold for *M*. *mucilaginosa* and 1.21–1.30-fold for *M*. *rubra* across the tested salinity levels (Fig. 10a; p < 0.05). In contrast, although POD, CAT, and APX activities tended to be higher in inoculated plants than in uninoculated plants at corresponding NaCl concentrations, these differences were not consistently statistically significant according to Tukey’s post hoc test (Fig. 10b–d). Two-way ANOVA revealed a significant main effect of salinity on all antioxidant enzymes, whereas the effects of inoculation and the salinity × inoculation interaction varied among enzymes. Overall, these results indicate that salinity is the dominant factor driving antioxidant enzyme responses in *N*. *tabacum*, while bacterial inoculation modulates specific components of the antioxidant system without uniformly enhancing all enzyme activities under salt stress.

### Hierarchical cluster heatmap

**Fig. 11:**
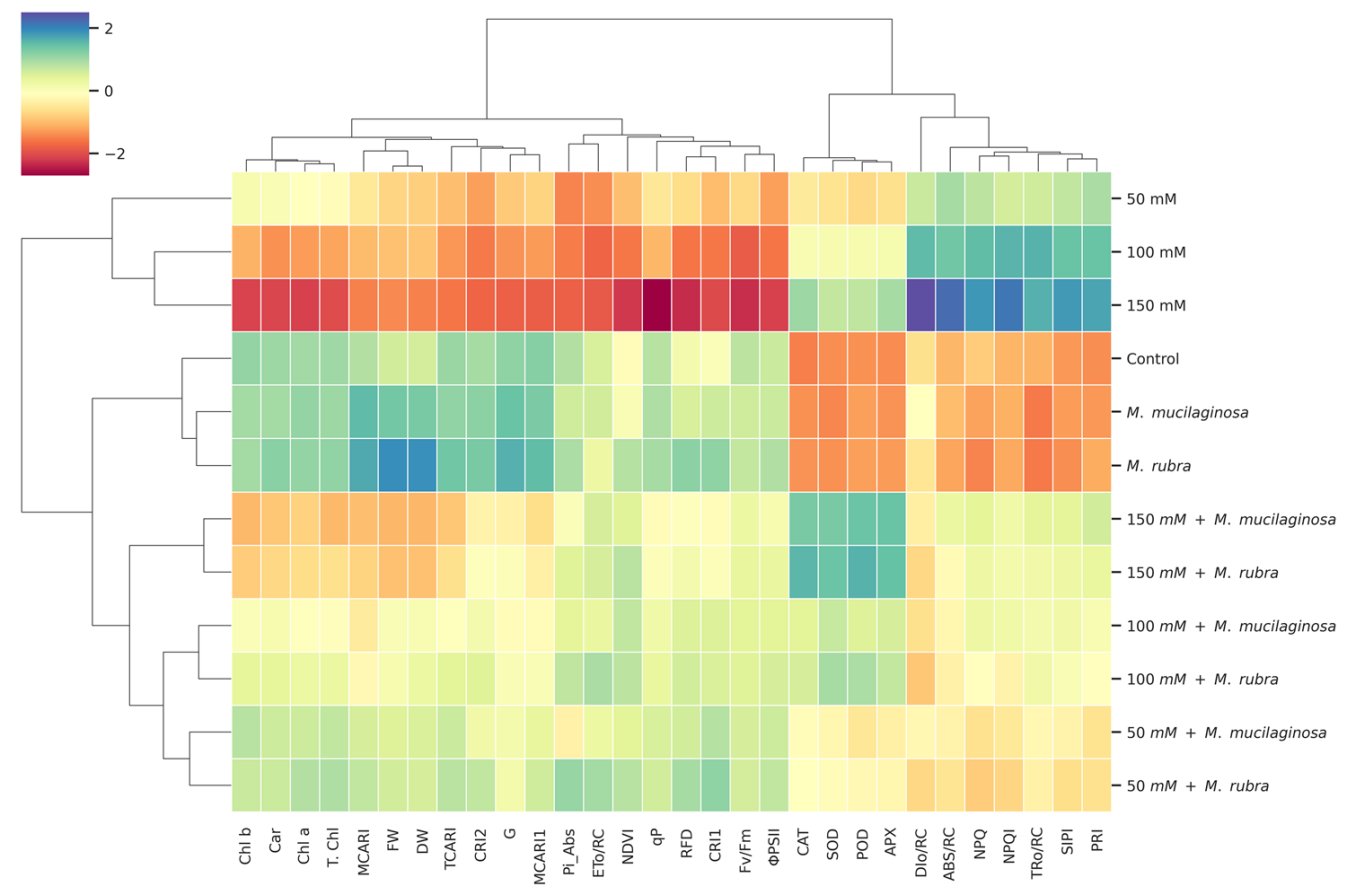
Hierarchical cluster heatmap displaying standardized Z-scores of 31 morphophysiological, spectral, chlorophyll fluorescence, and antioxidant traits in *N*. *tabacum* under different salinity conditions (0, 50, 100, and 150 mM NaCl) and bacterial inoculations (*M*. *mucilaginosa* and *M*. *rubra*). Rows represent treatments; columns represent parameters. Color gradients indicate low (red) to high (blue) normalized values. Cluster analysis was performed using Ward’s method and Euclidean distance

A hierarchical cluster heatmap (Fig. 11) was constructed to visualize the multivariate response of *N*. *tabacum* to salinity stress (50, 100, and 150 mM NaCl) and bacterial inoculation (*M*. *mucilaginosa* and *M*. *rubra*), integrating 31 morphophysiological, biochemical, and spectral traits. The heatmap revealed a clear separation between uninoculated salt-stressed plants and non-stressed or inoculated treatments, highlighting the combined influence of salinity and bacterial inoculation on plant performance. Growth traits, including fresh weight (FW) and dry weight (DW), clustered closely with control and inoculated treatments and showed progressively lower values under increasing salinity, particularly in uninoculated plants, with partial recovery observed following bacterial inoculation. This integrative approach was applied to capture coordinated physiological and biochemical responses rather than relying on individual traits in isolation. Photosynthetic efficiency parameters, such as Fv/Fm and Φ_PSII_, co-clustered with qP and RFD and were associated with non-stressed and inoculated treatments, whereas salt-stressed uninoculated plants grouped with parameters indicative of photochemical impairment. NPQ clustered inversely with Fv/Fm and Φ_PSII_ and was more closely associated with salt-stressed conditions, while inoculated treatments showed patterns consistent with moderated energy dissipation. Similarly, PI_ABS_ was lower under salinity stress and showed recovery trends in inoculated plants. OJIP-derived parameters (ABS/RC, TRo/RC, ETo/RC, and DIo/RC) exhibited distinct clustering patterns. Uninoculated salt-stressed plants grouped with higher ABS/RC and DIo/RC values, reflecting increased energy dissipation and reduced PSII functionality, whereas inoculated treatments were more closely associated with ETo/RC, suggesting improved electron transport efficiency. Spectral vegetation indices related to pigment content and canopy structure (MCARI, TCARI, MCARI1, CRI1, and CRI2) were reduced under salinity stress and clustered with indices linked to pigment degradation, while NDVI and PRI grouped with inoculated treatments, consistent with improved pigment stability and photosynthetic performance. Pigment contents (chlorophyll a, chlorophyll b, total chlorophyll, and carotenoids) clustered with inoculated and lower-salinity treatments, whereas uninoculated salt-stressed plants showed reduced pigment-associated clustering. Antioxidant enzymes (SOD, POD, CAT, and APX) clustered predominantly with inoculated and non-stressed treatments, whereas uninoculated salt-stressed plants grouped separately. Although *M*. *rubra* often showed slightly higher mean antioxidant activities at higher NaCl concentrations, clustering patterns indicated broadly comparable antioxidant responses between the two *Mokoshia* strains. Overall, the hierarchical clustering differentiates salinity-induced stress responses from bacterial-associated mitigation patterns, with both *M*. *mucilaginosa* and *M*. *rubra* showing coordinated associations across multiple physiological and biochemical traits.

### Principal component analysis (PCA)

**Fig. 12:**
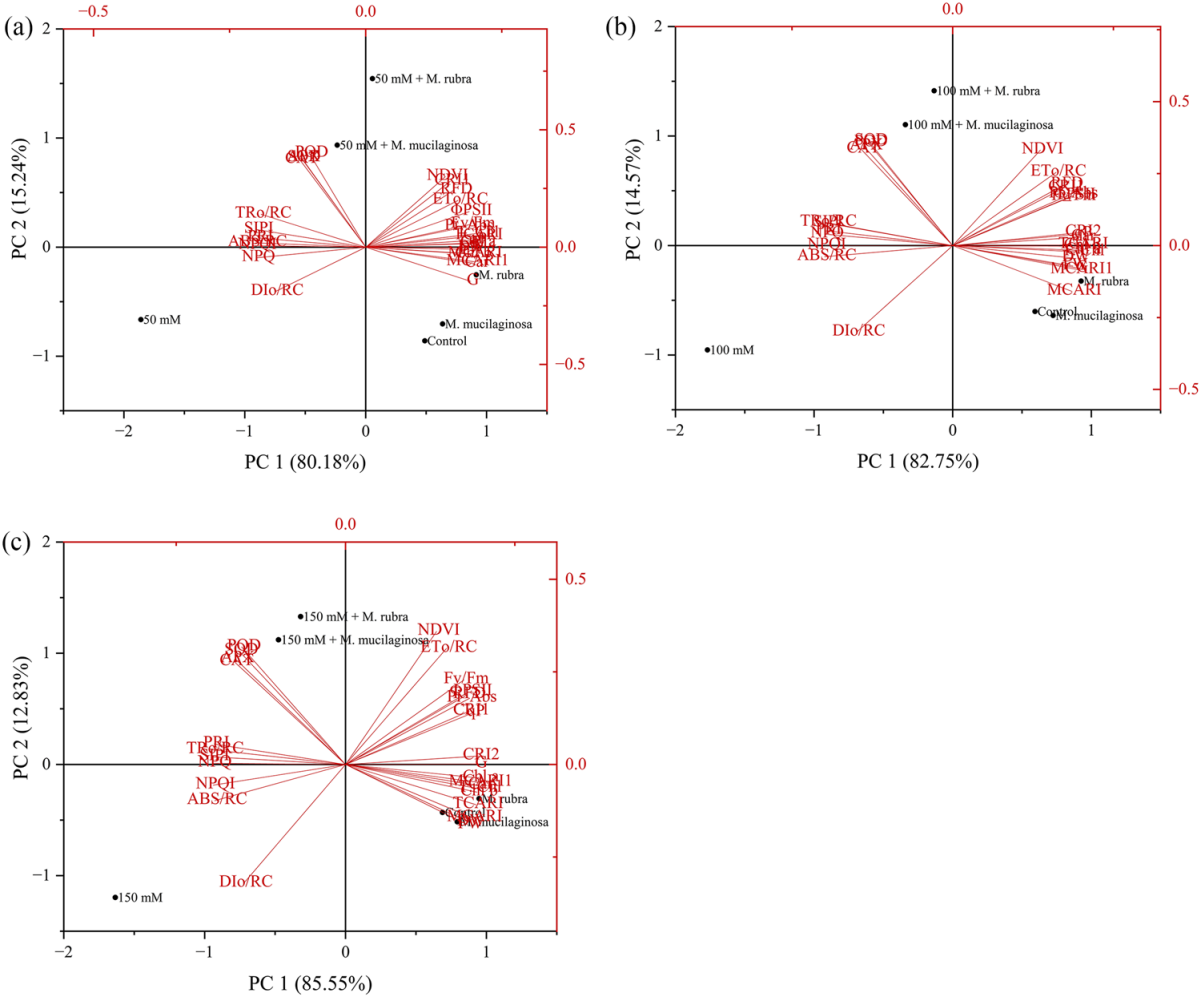
**a–c** Principal component analysis (PCA) biplots showing treatment distribution and associations with morphophysiological, biochemical, spectral, photosynthetic, and antioxidant traits in *N*. *tabacum* under **a** 50 mM, **b** 100 mM, and **c** 150 mM NaCl stress. Parameters include FW, DW, Fv/Fm, Φ_PSII_, NPQ, qP, RFD, Pi_Abs, energy fluxes (ABS/RC, TRo/RC, ETo/RC, DIo/RC), spectral indices (NDVI, PRI, SIPI, CRI1, CRI2, TCARI, MCARI, MCARI1), pigments (Chl a, Chl b, T. Chl, Car), and antioxidant enzymes (SOD, POD, CAT, APX). Treatments include NaCl levels with or without *M*. *mucilaginosa* or *M*. *rubra* inoculation. Inoculated treatments clustered with traits associated with improved physiological performance, while uninoculated salt-stressed treatments aligned with stress-related parameters

Principal component analysis (PCA) was performed to evaluate multivariate patterns among physiological, biochemical, and spectral parameters in *N*. *tabacum* under 50, 100, and 150 mM NaCl stress, with or without inoculation by *M*. *mucilaginosa* and *M*. *rubra*. At 50 mM NaCl (Fig. 12a), PCA explained 95.42% of the total variance, with PC1 and PC2 accounting for 80.18% and 15.24%, respectively. Inoculated treatments were separated from the uninoculated treatment along PC1. Fresh weight (FW) and dry weight (DW) loaded positively and were associated with inoculated treatments. Photosynthetic parameters, including Fv/Fm, Φ_PSII_, qP, and RFD, as well as energy flux parameters (PI_ABS_, ABS/RC, TRo/RC, and ETo/RC), clustered closer to inoculated groups, whereas DIo/RC aligned with the uninoculated 50 mM NaCl treatment. Antioxidant enzymes (SOD, POD, CAT, and APX) loaded primarily along PC2. Spectral indices (NDVI, G, MCARI, MCARI1, TCARI, CRI1, CRI2, SIPI, NPQI, and PRI) and pigment traits (chlorophyll a, chlorophyll b, total chlorophyll, and carotenoids) also showed associations with inoculated treatments. At 100 mM NaCl (Fig. 12b), PCA accounted for 97.32% of the total variance, with PC1 explaining 82.75% and PC2 explaining 14.57%. Inoculated treatments clustered separately from the uninoculated 100 mM NaCl treatment. FW, DW, NDVI, Fv/Fm, Φ_PSII_, ETo/RC, and CRI2 loaded positively and were associated with inoculated treatments. In contrast, DIo/RC, NPQ, NPQI, and ABS/RC were associated with the uninoculated 100 mM NaCl treatment. Antioxidant enzyme activities and pigment-related parameters grouped closer to inoculated treatments. At 150 mM NaCl (Fig. 12c), PCA explained 98.38% of the total variance, with PC1 and PC2 accounting for 85.55% and 12.83%, respectively. Inoculated treatments clustered separately from the uninoculated 150 mM NaCl treatment. Parameters such as NDVI, ETo/RC, Fv/Fm, Φ_PSII_, CRI1, CRI2, RFD, and pigment contents were associated with inoculated treatments, whereas DIo/RC, NPQ, NPQI, TRo/RC, and ABS/RC aligned with the uninoculated treatment. Antioxidant enzyme activities remained closely associated with inoculated groups across salinity levels. Additional Pearson correlation analyses supporting these multivariate relationships are provided in the Supplementary Materials (Fig. S8a–b).

## Discussion

Soil salinity is an increasingly severe constraint to global agriculture, particularly in marginal and cold-prone environments where salt accumulation and low temperatures jointly exacerbate plant stress responses. Salinity disrupts plant water relations, ion homeostasis, photosynthetic processes, and cellular redox balance, ultimately resulting in reduced biomass accumulation and crop productivity (Shirokikh et al. 2022; Pecherina et al. 2022). Although genetic improvement and agronomic management have been explored to mitigate salinity stress, these approaches are often time-consuming, costly, and inconsistent under field conditions. In this context, plant growth–promoting bacteria (PGPB) have emerged as an effective and sustainable alternative due to their capacity to enhance stress tolerance through multiple, synergistic physiological and biochemical mechanisms (Numan et al. 2018; Kour & Yadav 2023; Xie et al. 2024).

Salinity-induced growth inhibition in plants is primarily driven by osmotic stress, ion toxicity, and hormonal imbalance, which collectively restrict carbon assimilation and cell expansion (Mishra et al. 2023). In line with these mechanisms, uninoculated *N*. *tabacum* plants exhibited pronounced reductions in fresh and dry biomass under increasing NaCl concentrations. In contrast, inoculation with *Mokoshia* spp. improved biomass accumulation, with effects that were more pronounced at lower salinity levels and diminished under higher NaCl concentrations. Similar growth-promoting effects of PGPB under salt stress have been widely reported in rice, tomato, and soybean, where bacterial inoculation enhanced root development, ion uptake, and overall stress resilience (Khan et al. 2019, 2021; Kumar et al. 2021; Sánchez et al. 2023).

The observed biomass improvement can be attributed to the possession of multiple PGP traits by *Mokoshia* spp., including indole-3-acetic acid production, phosphate solubilization, siderophore synthesis, and nitrogen fixation. These traits collectively promote root elongation, improve nutrient acquisition, and support metabolic activity under saline conditions (Ha-Tran et al. 2021; Kumar et al. 2022). Such multifunctional mechanisms are particularly important under salinity, where nutrient availability and hormonal signaling are severely disrupted.

Photosynthetic inhibition is one of the earliest and most damaging consequences of salinity stress, especially in salt-sensitive species such as *N*. *tabacum* (Pecherina et al. 2022). Excessive Na⁺ accumulation interferes with PSII structure, reduces electron transport efficiency, and increases energy dissipation, leading to photoinhibition and oxidative damage (Stefanov et al. 2024). In the present study, salt stress markedly impaired PSII efficiency and increased non-photochemical energy dissipation in uninoculated plants. *Mokoshia* inoculation was associated with attenuation of these effects, consistent with improved photochemical performance and regulation of light energy use under salinity.

These findings are consistent with previous studies demonstrating that PGPB can protect PSII functionality under salinity by maintaining chlorophyll content, stabilizing thylakoid membranes, and improving ion homeostasis (Rossi et al. 2021; Yaghoubi Khanghahi et al. 2021; Gupta et al. 2022). The ability of *Mokoshia* spp. to sustain PSII efficiency under high NaCl concentrations suggests that psychrotolerant bacteria may be particularly effective in preserving photosynthetic machinery under combined stress conditions.

Fast chlorophyll fluorescence (OJIP) analysis provided deeper insight into the structural and functional integrity of PSII under salinity stress. Salinity typically increases absorbed and dissipated energy fluxes per reaction center while reducing electron transport efficiency, reflecting inactivation of PSII centers and impaired connectivity (Strasser et al. 2004; Guidi et al. 2019). Inoculated plants exhibited moderated energy dissipation and sustained electron transport, indicating improved PSII stability.

Spectral reflectance indices provided complementary, non-destructive indicators of plant physiological status under salinity. Declines in vegetation indices associated with chlorophyll content and canopy greenness are commonly observed under salt stress and reflect pigment degradation and reduced photosynthetic capacity (Vennam et al. 2025). *Mokoshia* inoculation significantly mitigated these declines, indicating improved pigment stability and canopy vitality.

Direct pigment analysis confirmed that salinity-induced losses of chlorophylls and carotenoids were substantially alleviated by bacterial treatment. Chlorophyll preservation is critical for maintaining light-harvesting efficiency, while carotenoids play a central role in photoprotection and reactive oxygen species scavenging (Fan et al. 2020). Enhanced pigment retention in *Mokoshia*-inoculated plants aligns with previous reports showing that PGPB protect pigment biosynthesis pathways and limit oxidative degradation under salinity (Singh & Jha 2017; Khan et al. 2019; Lee et al. 2024).

Salinity stress inevitably leads to excessive production of reactive oxygen species, triggering the activation of antioxidant enzymes such as SOD, POD, CAT, and APX as part of an intrinsic defense response (Fukami et al. 2017; Gupta et al. 2022). In agreement with these reports, salt stress significantly induced antioxidant enzyme activities in uninoculated *N*. *tabacum* plants. Importantly, Mokoshia inoculation did not suppress this response but modulated it and, in some cases, provided additional enhancement.

Although the magnitude of enzyme induction was not always significantly higher in inoculated plants compared with uninoculated salt-stressed plants, bacterial treatment was consistently associated with improved coordination of antioxidant activity alongside enhanced photosynthetic performance. This suggests that *Mokoshia* spp. contribute to more efficient redox regulation rather than merely amplifying stress-induced enzyme activity, a phenomenon previously reported for other PGPB–plant interactions under salinity (Rossi et al. 2021; Gupta et al. 2021).

The integration of physiological, biochemical, and spectral traits through multivariate analyses provided a comprehensive framework for evaluating plant responses to salinity and bacterial inoculation. Such approaches are increasingly recognized as essential for capturing coordinated stress responses that cannot be resolved by single traits alone. The consistent separation between uninoculated salt-stressed plants and *Mokoshia*-treated plants across clustering and ordination analyses supports a consistent association between bacterial inoculation and coordinated physiological responses under salinity stress.

Collectively, these findings suggest that psychrotolerant Antarctic *Mokoshia* spp. confer multifaceted protection against salinity stress by stabilizing photosynthetic processes, preserving pigment integrity, and optimizing antioxidant defenses. Their effectiveness under saline conditions, combined with their adaptation to low temperatures, highlights their potential as bioinoculants for sustainable agriculture in cold and marginal environments. To our knowledge, this study provides the first evidence that members of the genus *Mokoshia* enhance salt tolerance in plants through coordinated regulation of photosynthetic and antioxidant systems, expanding the biotechnological relevance of Antarctic-derived PGPB. In this study, salinity stress was imposed using NaCl, which is widely employed as an agronomically relevant model to simulate saline conditions in plants and allows direct comparison with previous studies. We acknowledge that other salts (e.g., KCl, CaCl₂, MgCl₂) may impose partially distinct ionic effects, and future studies will be required to determine whether *Mokoshia*-mediated stress alleviation extends across different salt chemistries. The present study focused on plant physiological responses to bacterial inoculation rather than direct quantification of bacterial colonization. Although bacterial persistence or colonization was not assessed using molecular approaches such as qPCR, the consistent improvements in plant growth, photosynthetic performance, pigment stability, and antioxidant regulation demonstrate a clear functional response to bacterial treatment. These effects may arise from direct plant–microbe interactions in the rhizosphere or from indirect mechanisms, including enhanced nutrient availability and modification of the soil microenvironment. Distinguishing between these modes of action will be an important focus of future studies.

### Conclusion and future perspectives

This study provides evidence that the Antarctic-origin psychrotolerant strains *M*. *mucilaginosa* and *M*. *rubra* alleviate the adverse effects of salinity stress in *Nicotiana tabacum*. Inoculated plants exhibited improved growth, stabilized photosynthetic performance, preserved pigment integrity, and modulated antioxidant defenses, with both *Mokoshia* strains contributing similarly to enhanced stress tolerance under saline conditions. The presence of multiple plant growth–promoting traits further highlights the capacity of these bacteria to support host performance across increasing NaCl levels. Collectively, the findings indicate that *Mokoshia* spp. represent promising and sustainable bioinoculants for crops cultivated in salinity-affected and cold-prone regions. Future research should prioritize field validation across diverse crop species and agroecosystems to confirm the consistency of these effects under realistic environmental conditions. Integrative molecular approaches, including genomics, transcriptomics, and proteomics, will be essential to elucidate the signaling pathways and microbial determinants underlying stress mitigation. In parallel, efforts focused on inoculant formulation, shelf-life optimization, and ecological risk assessment will be critical to facilitate safe and effective translation from laboratory studies to agricultural practice. Harnessing the biotechnological potential of *Mokoshia* spp. offers a pathway toward developing climate-resilient and sustainable farming systems capable of addressing the escalating challenge of soil salinity.

## Supplementary Information

Below is the link to the electronic supplementary material.

Supplementary file1 (DOCX 5817 KB)

## Data Availability

The data supporting the findings of this study are available from the corresponding author upon reasonable request.
