# Supplementary material for: Psychrotolerant antarctic Mokoshia mucilaginosa and Mokoshia rubra enhance salt Stress tolerance in Nicotiana tabacum via photosynthetic stabilization and antioxidant regulation
Source: Plant Cell Rep. 2026 Feb 21;45(3):61. doi: 10.1007/s00299-026-03732-w (PMC12924858; doi:10.1007/s00299-026-03732-w)
Supplement: Supplementary file 1 — Supplementary file1 (DOCX 5817 KB) [file 299_2026_3732_MOESM1_ESM.docx]

**Plant Growth-Promoting Traits of *M*. *mucilaginosa* and *M*. *rubra***

Both *M*. *mucilaginosa* and *M*. *rubra* exhibited positive activity in multiple plant growth-promoting (PGP) traits. Specifically, both strains demonstrated the ability to produce indole-3-acetic acid (IAA), solubilize phosphate, produce siderophores, and fix nitrogen (Supplementary Table 1).

| **Strains** | **IAA**  **(µg mL⁻¹)** | **Phosphate Solubilization**  **(halo diameter, mm)** | **Siderophore Production**  **(CAS halo, mm)** | **Nitrogen Fixation**  **(growth score)** |
| --- | --- | --- | --- | --- |
| *M*. *mucilaginosa* | 18.6 ± 1.2 | 9.4 ± 0.6 | 7.8 ± 0.5 | Positive growth |
| *M*. *rubra* | 22.1 ± 1.5 | 11.2 ± 0.8 | 9.6 ± 0.7 | Positive growth |

**Supplementary Table 1:** Evaluation of plant growth-promoting traits in *M*. *mucilaginosa* and *M*. *rubra*. Values are means ± SD (n = 5). Nitrogen fixation potential was assessed qualitatively based on growth on nitrogen-free semi-solid medium.

**Chlorophyll fluorescence imaging of *N*. *tabacum*** **leaves under NaCl stress and bacterial inoculation treatments.**

Chlorophyll fluorescence imaging of *Nicotiana tabacum* leaves was used to provide a qualitative visualization of spatial variation in photosynthetic parameters under NaCl stress and bacterial inoculation (Figs. S1–S5). Across all parameters, salinity stress was associated with visible changes in color distribution relative to non-stressed controls, whereas inoculated plants generally exhibited intermediate or distinct color patterns compared with uninoculated stressed plants. As leaf size varied due to salinity-induced growth effects, fluorescence imaging was employed solely as a supportive, qualitative tool rather than for quantitative comparison. Accordingly, these images illustrate general trends in PSII-related fluorescence parameters and should be interpreted in conjunction with the quantitative chlorophyll fluorescence data presented in the main manuscript. Fluorescence images are presented for qualitative visualization only and were not subjected to statistical analysis.


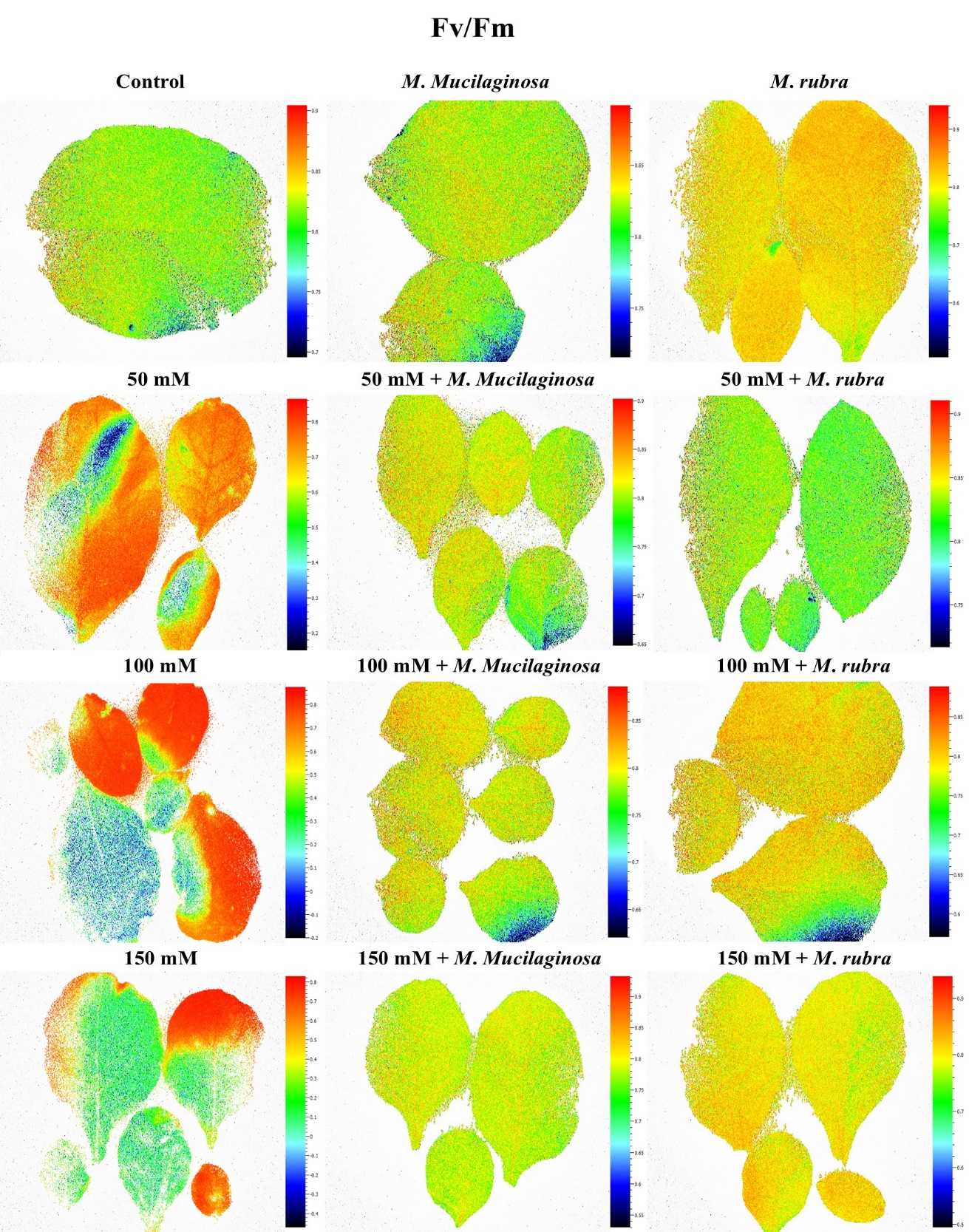


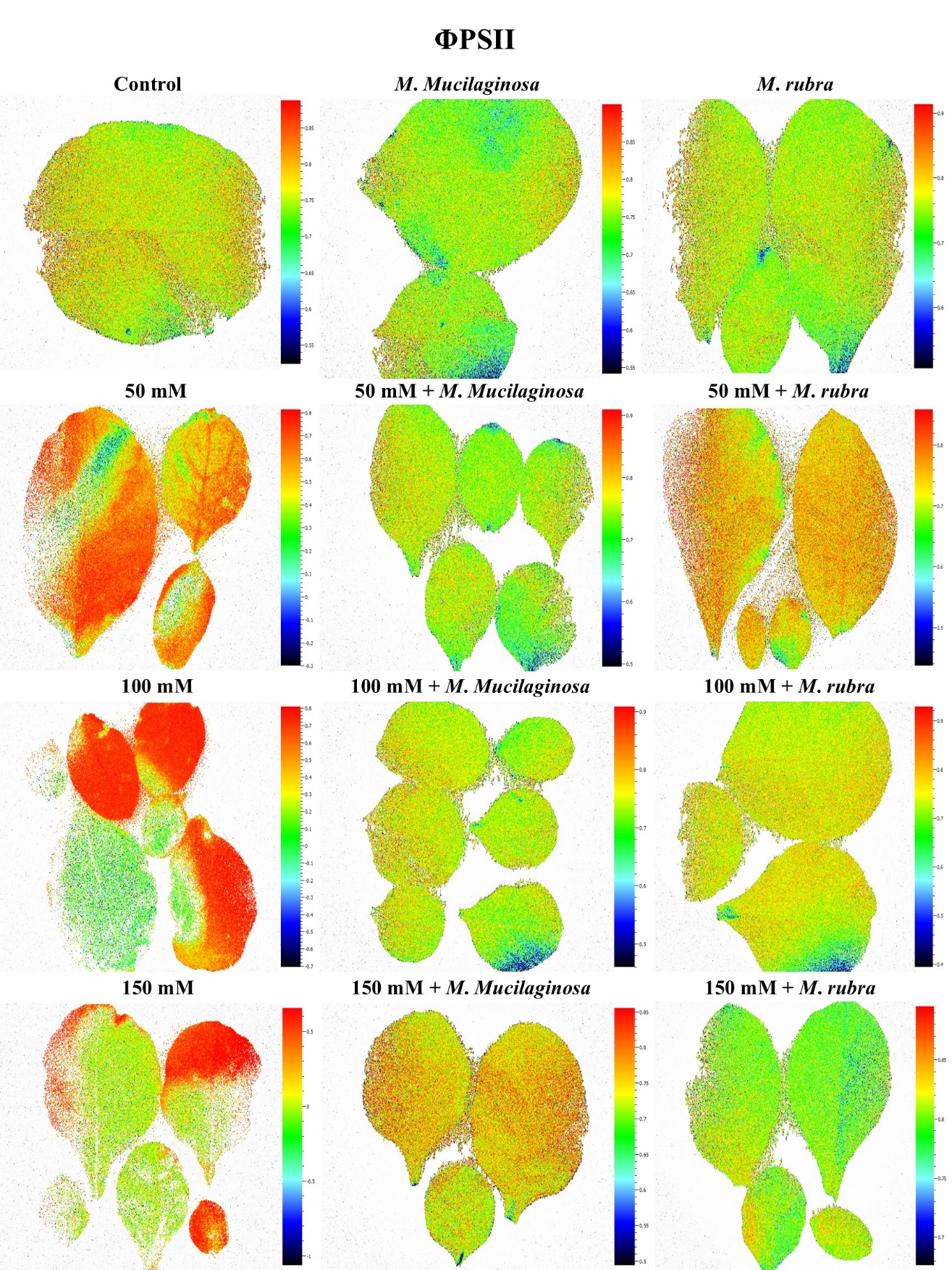


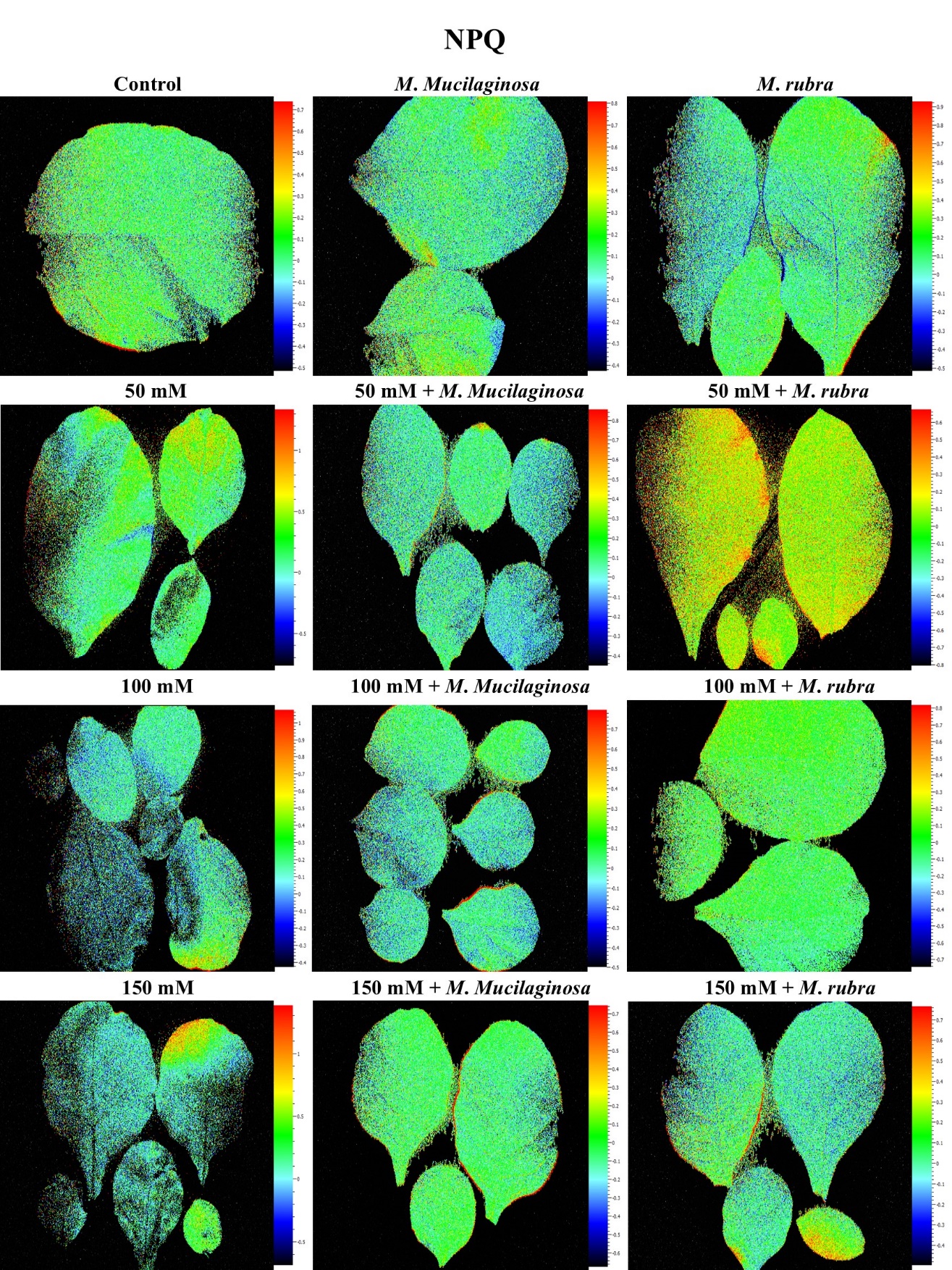
c


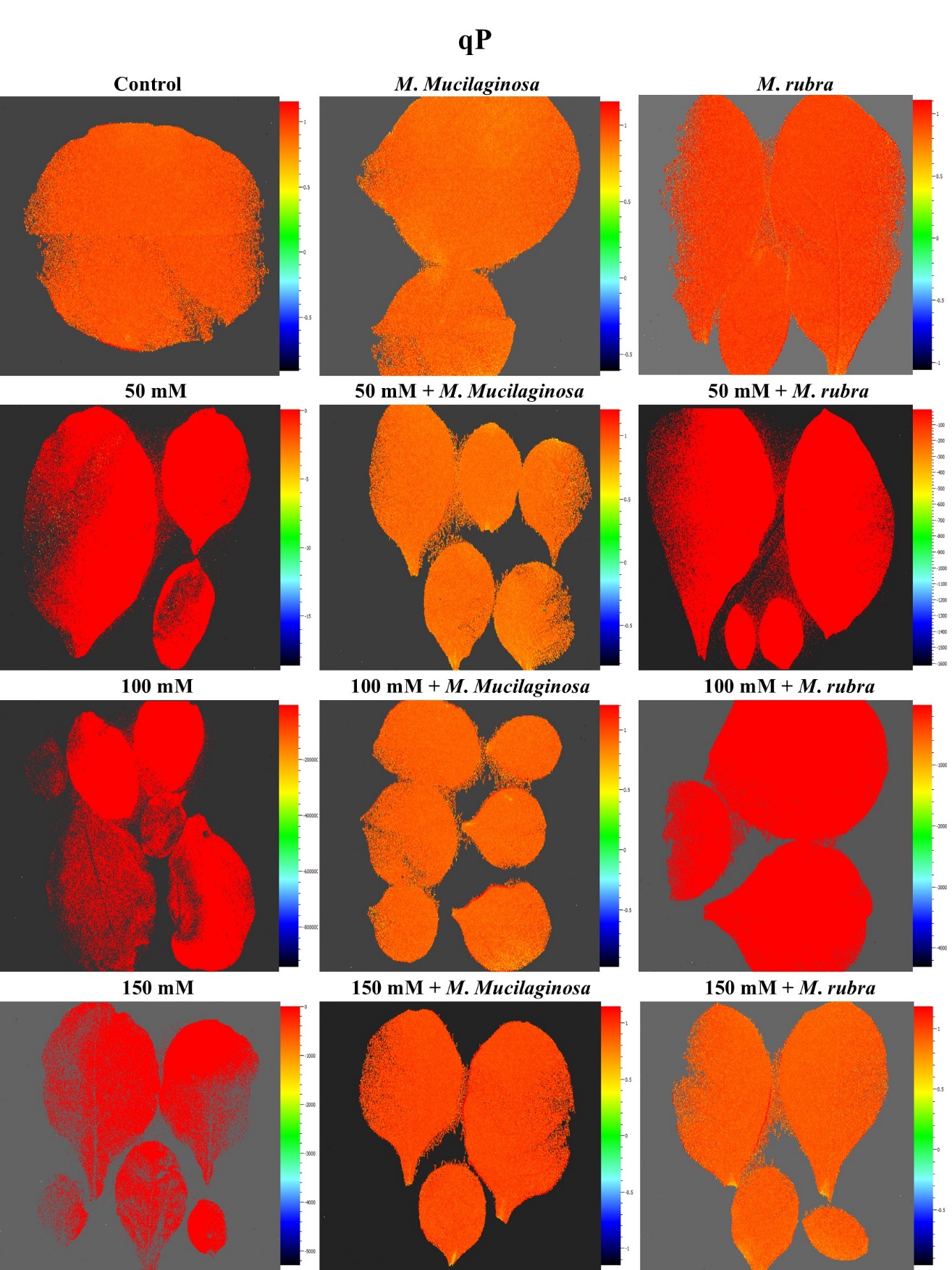


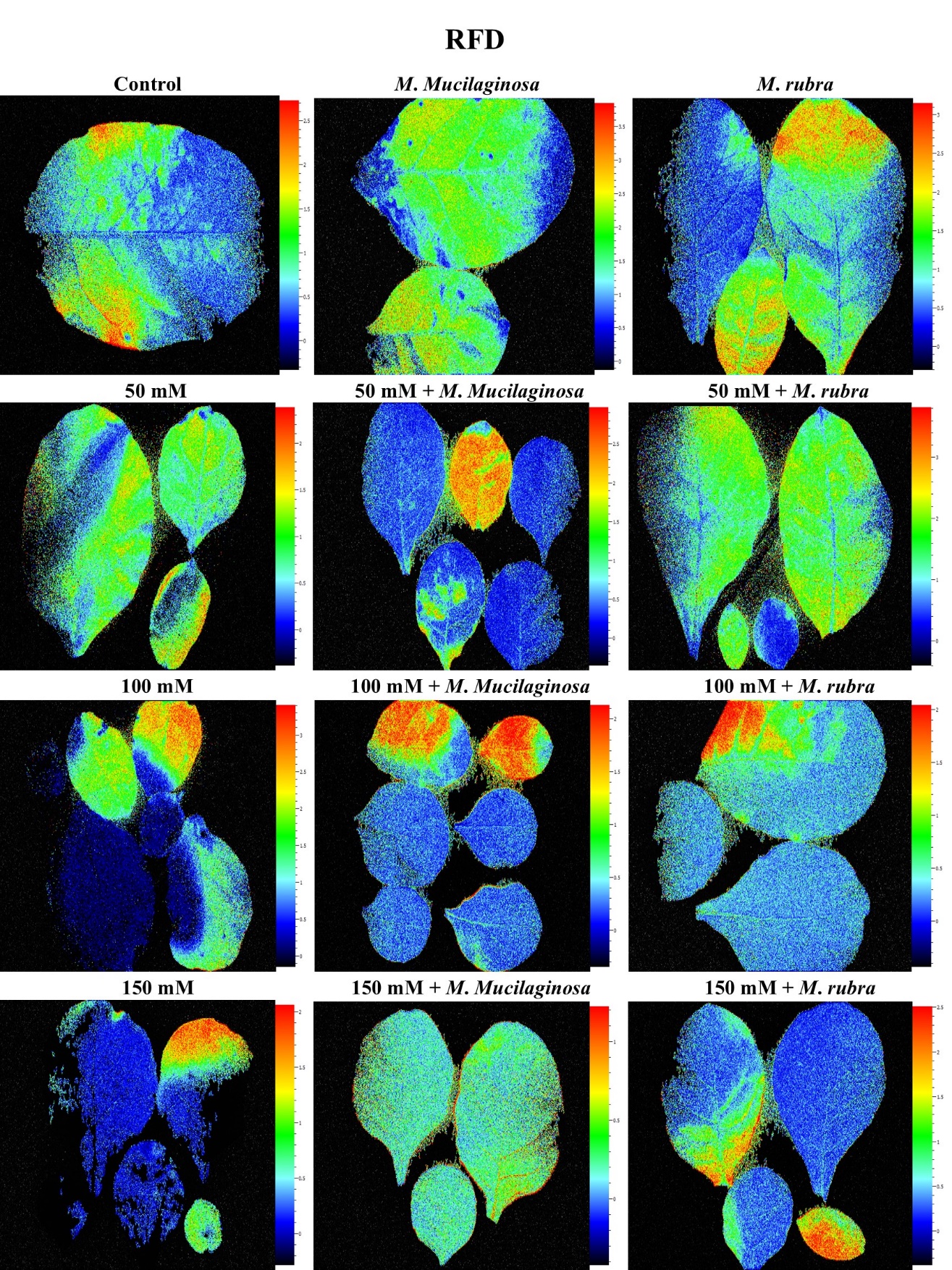


**Supplementary Figure S1–S5:** False-color FluorCam images visualize the spatial distribution of five chlorophyll fluorescence parameters—(a) Fv/Fm, (b) Φ_PSII_, (c) NPQ, (d) qP, and (e) RFD—in *N*. *tabacum* leaves exposed to NaCl concentrations of 0, 50, 100, and 150 mM. Each treatment was assessed in uninoculated plants and those inoculated with *M*. *mucilaginosa* and *M*. *rubra*. Color scales represent pixel-level fluorescence values from low (blue) to high (red/yellow), specific to each parameter. Green-to-yellow tones indicate healthy photochemical performance, whereas shifts to red, blue, or black reflect stress-induced photoinhibition or inefficiencies. Each image is representative of the mean spatial signal across biological replicates per treatment.

**Normalized OJIP Transients**

Normalized chlorophyll a fluorescence transients (OJIP curves) illustrate the effects of NaCl stress and bacterial inoculation on PSII behavior in *N*. *tabacum* (Fig. S6). In uninoculated plants, increasing NaCl concentrations were associated with progressive suppression of the O–P rise. Inoculated plants exhibited altered OJIP profiles relative to uninoculated controls under salinity stress, with both *M*. *mucilaginosa* and *M*. *rubra* treatments showing deviations from stress-induced patterns. These data are provided as supplementary visualization of PSII response patterns and should be interpreted alongside the quantitative OJIP parameters reported in the main text.


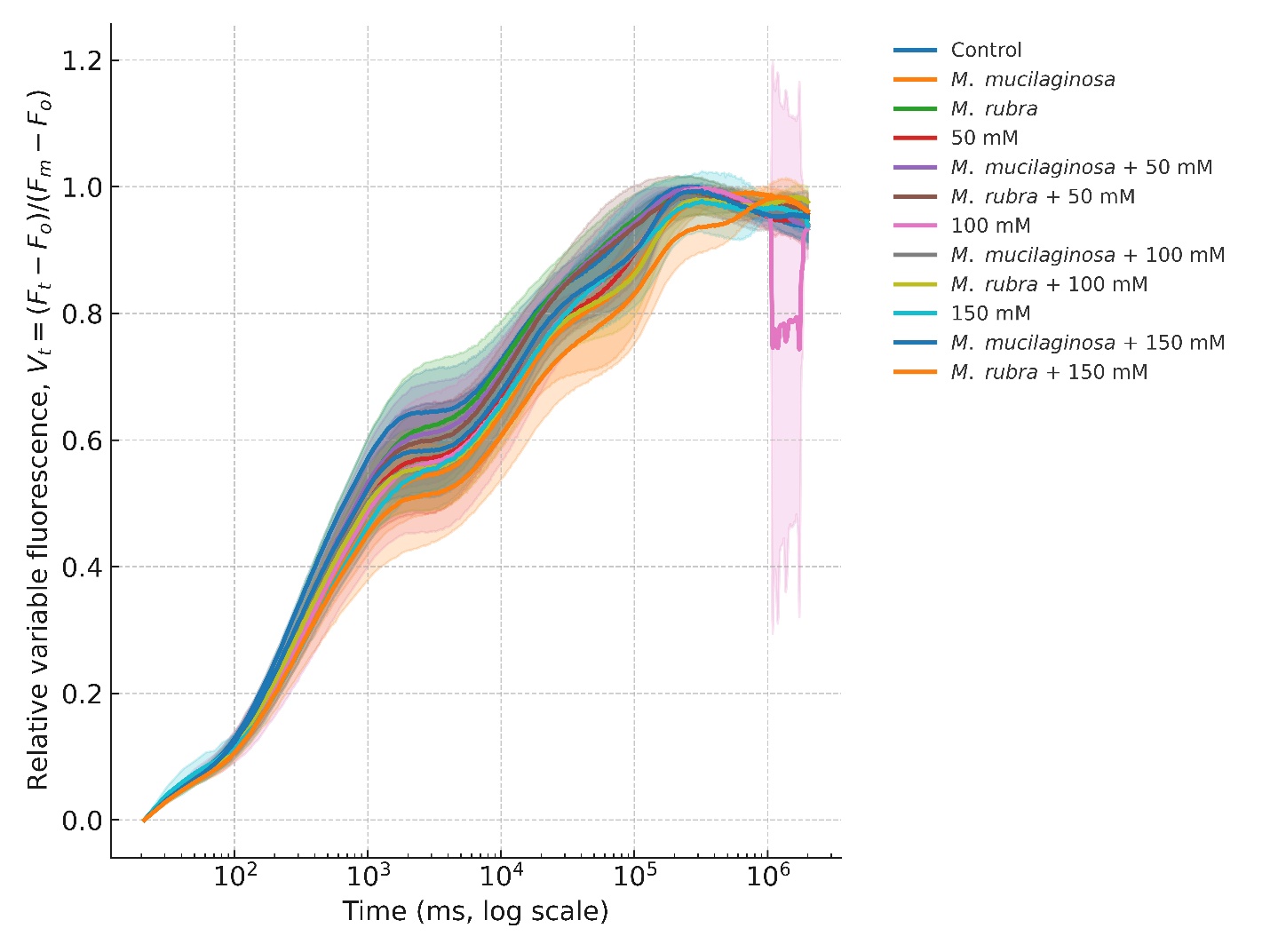


**Supplementary Figure S6.** Normalized chlorophyll a fluorescence transient (OJIP curves) of Nicotiana tabacum under salt stress and bacterial inoculation. Curves represent mean ± SD of normalized fluorescence (Ft − Fo) / (Fm−Fo) on a logarithmic time scale (µs). Treatments include control, bacterial inoculation (M. mucilaginosa, M. rubra), NaCl stress (50, 100, 150 mM), and combined stress with inoculation (n = 5).

**L- and K-band Analyses**

Differential L- and K-band analyses illustrate changes in PSII-related fluorescence features under salinity stress and bacterial inoculation (Fig. S7). In uninoculated plants, increased band amplitudes were observed with rising NaCl concentrations, whereas inoculated treatments showed altered band patterns relative to uninoculated controls. These analyses provide supplementary visualization of PSII donor- and acceptor-side responses and support the quantitative fluorescence parameters presented in the main manuscript.


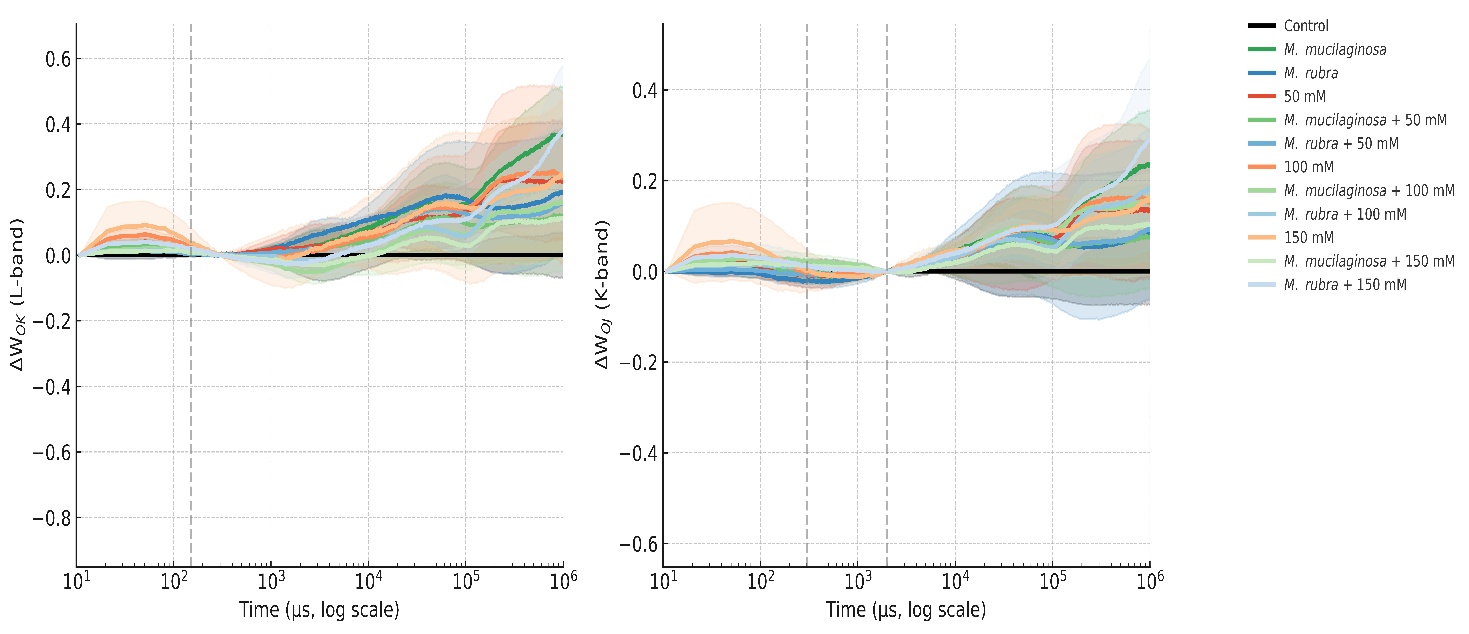


**Supplementary Figure S7.** Differential L- and K-band analyses of chlorophyll a fluorescence in Nicotiana tabacum under salt stress and bacterial inoculation. Left panel: ΔW_OK_ (L-band) calculated as the difference from control at ~150 µs. Right panel: ΔW_OJ_ (K-band) calculated as the difference from control at ~300–2000 µs. Curves represent mean ± SD (n = 5) differences relative to control (black baseline at zero) on a logarithmic time scale (µs). Vertical dashed lines indicate characteristic L- and K-band timepoints. Treatments include control, bacterial inoculation (*M*. *mucilaginosa*, M. *rubra*), NaCl stress (50, 100, 150 mM), and inoculation with *M*. *mucilaginosa* or *M*. *rubra* in combination with NaCl.

**Pearson correlation heatmaps**

The Pearson correlation heatmaps (Fig. S8a–b) summarize pairwise relationships among growth traits, chlorophyll fluorescence parameters, energy flux variables, vegetation and spectral indices, pigment contents, and antioxidant enzyme activities under inoculation with *M*. *mucilaginosa* (a) and *M*. *rubra* (b). The heatmaps illustrate patterns of positive and negative correlations among measured variables, providing an overview of trait interrelationships within each inoculation treatment. These correlations are descriptive and exploratory in nature and are intended to complement the univariate analyses and multivariate ordination (PCA) presented in the main manuscript, without implying causal relationships.


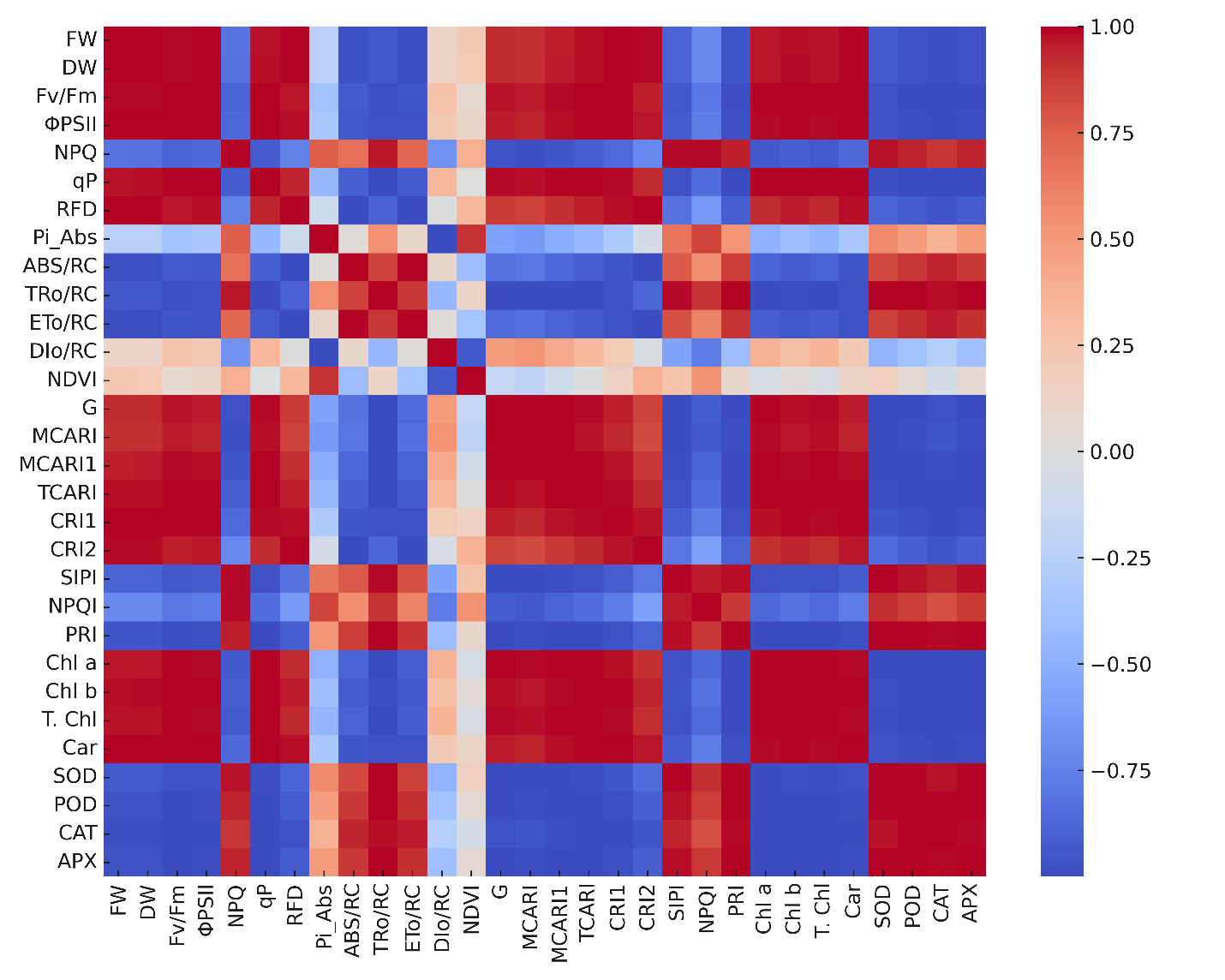


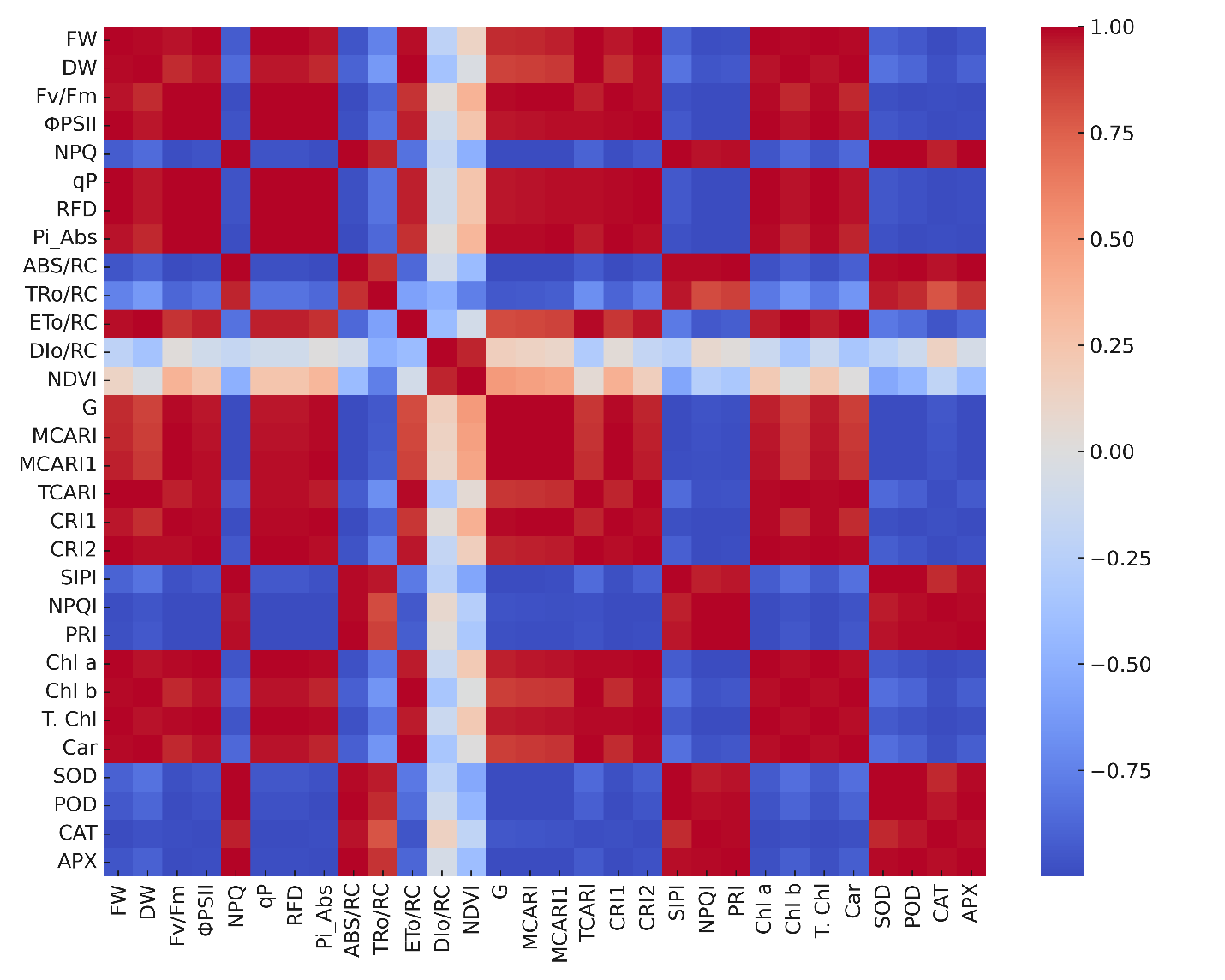


**Supplementary Figure S8a–8b.** Pearson correlation heatmaps showing pairwise relationships among growth traits, chlorophyll fluorescence, energy flux parameters, vegetation and spectral indices, pigments, and antioxidant enzymes in *N*. *tabacum* inoculated with (a) *M*. *mucilaginosa* and (b) *M*. *rubra*. The color scale represents correlation coefficients (r), with red indicating strong positive and blue indicating strong negative correlations.
